# Supplementary material for: TUG1-mediated R-loop resolution at microsatellite loci as a prerequisite for cancer cell proliferation
Source: Nat Commun. 2023 Aug 22;14:4521. doi: 10.1038/s41467-023-40243-8 (PMC10444773; doi:10.1038/s41467-023-40243-8)
Supplement: Supplementary file 1 — Supplementary information [file 41467_2023_40243_MOESM1_ESM.pdf]

Supplementary information

Suzuki et al.

Supplementary Figure 1

Supplementary Figure 2

Supplementary Figure 3

Supplementary Figure 4

Supplementary Figure 5

Supplementary Figure 6

Supplementary Figure 7

Supplementary Figure 8

Supplementary Figure 9

Supplementary Figure 10

Supplementary Figure 11

Supplementary Figure 12

Supplementary Table 1. Summary of super resolution microscopic analysis

Supplementary Table 2. Top proteins identified by mass spectrometry from excised bands around 140 kDa

Supplementary Table 3. Median replication speeds measured by a DNA fiber assay

Supplementary Table 4. Genomic annotation of peaks differentially altered by TUG1 KD or CPT treatment, defined by homer

Supplementary Table 5. List of abbreviations

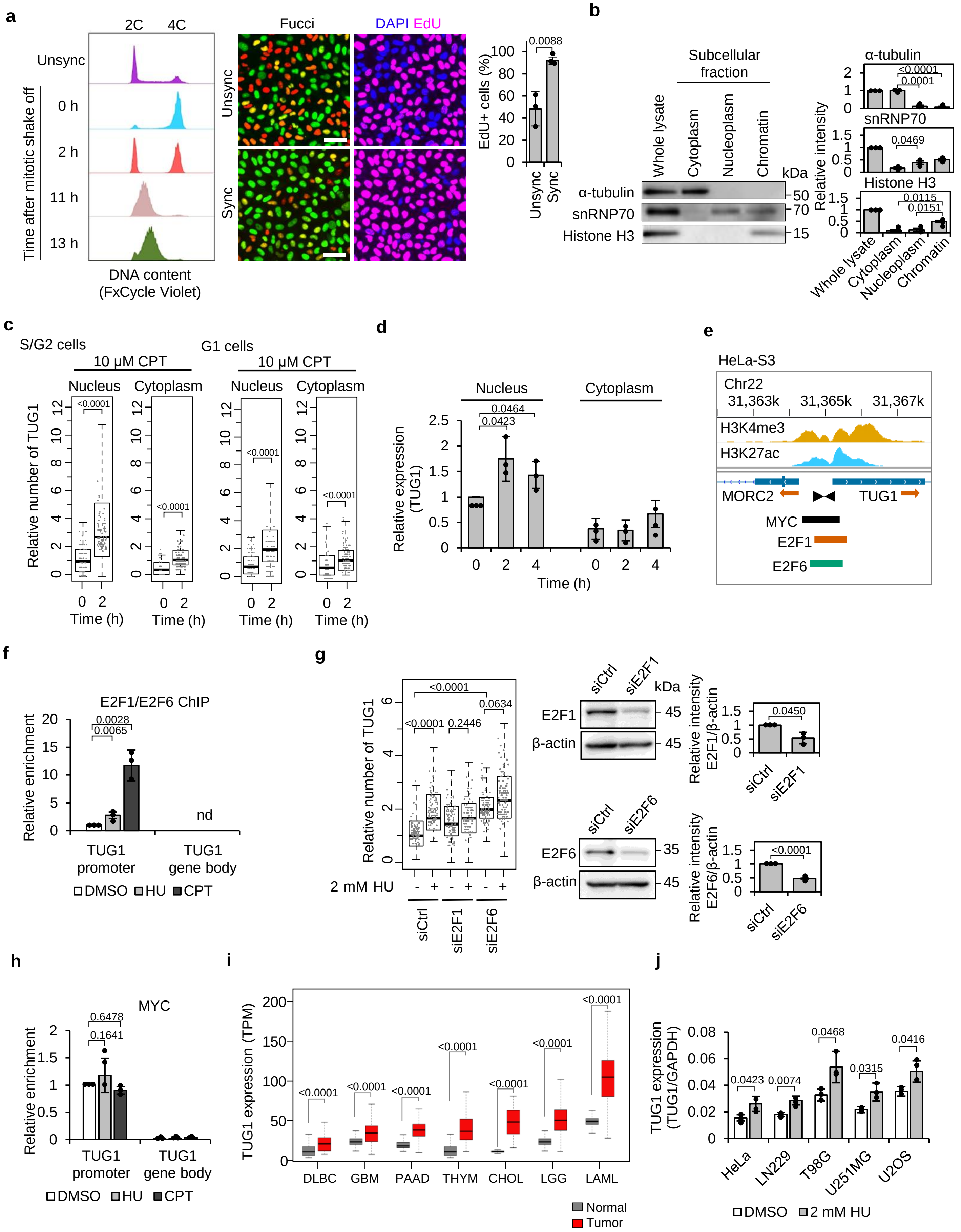

Supplementary Fig. 1.
ATR-CHK1-E2F pathway upregulates TUG1 expression in response to RS

Supplementary Fig. 1. ATR-CHK1-E2F pathway upregulates TUG1 expression in response to RS
a Almost all HeLa/Fucci2 cells collected by mitotic shake off were in the early S phase after 13 h. Left, cell-cycle distribution of synchronized cells shown by FCM profile. 2C and 4C indicate non-replicated and replicated genomes, respectively. Unsync: unsynchronized cells. Middle, an image of synchronized HeLa/Fucci2 cells at 13 h after mitotic shake off. Fluorescent ubiquitination-based cell cycle indicator (Fucci) visualized cell cycle progression; G1 cells and S/G2 cells are shown in red and green fluorescence, respectively. Sync: synchronized cells at 13 h after mitotic shake off. EdU-positive S phase cells were highly enriched by synchronization. Scale bar = 50 µm. Right, percentage of EdU-positive cells. Data are mean ± SD, n = 3. Two-sided *t*-test. **b** Left, validation of the subcellular fractionation by Western blotting with α-tubulin (a cytoplasmic marker), snRNP70 (a nucleoplasmic marker), and histone H3 (a chromatin marker). Right, bar graph generated by quantifying the Western blot. Values are normalized to the Whole lysate and represent the mean ± SD, n = 3. Two-sided *t*-test. **c** Relative number of TUG1 smFISH spots in the nucleus and the cytoplasm in HeLa/Fucci2 cells with 0 h and 2 h of 10 µM CPT treatment. Values are relative to the median number of TUG1 spots in the nucleus at 0 h. In the box plot, center lines show medians; box limits indicate the 25th and 75th percentiles; whiskers extend 1.5 times the interquartile range from the 25th and 75th percentiles. More than 70 cells were analyzed per sample. The two-sided Wilcoxon rank sum test was used for statistical analysis. The experiments were conducted in triplicate with similar results. **d** RT-qPCR analyses of the nucleus and cytoplasmic TUG1 in HeLa/Fucci2 cells. Synchronized S phase cells collected after 13 h of mitotic shake off were treated with 2 mM HU for 0, 2, 4 h, and subjected to subcellular fractionation. Values are relative to the TUG1 expression in the nucleus at 0 h. Data are mean ± SD, n = 3. Two-sided *t*-test. **e** Genome browser view of ENCODE ChIP-seq signal for H3K4me3 and H3K27ac in HeLa-S3 cells at the promoter of TUG1. Arrowheads indicate primer positions for ChIP-qPCR at TUG1 promoter. ENCODE ChIP-seq narrow peak of MYC, E2F1, and E2F6, in HeLa-S3 cells overlap the TUG1 transcriptional start site (GRCh37/hg19 chr22: 31365197). **f** ChIP-qPCR in HeLa/Fucci2 cells, treated with DMSO, 2 mM HU, or 10 µM CPT for 2 h. Relative occupancies of E2F1 to E2F6 at TUG1 promoter and gene-body region are shown. Data are mean ± SD, n = 3. Two-sided *t*-test. nd, not detected. **g** Left, relative number of TUG1 smFISH spots detected in siRNA-treated HeLa/Fucci2 cells. The indicated siRNAs were transfected for 48 h prior to 2 mM HU treatment for 2 h. Values are relative to the median number of TUG1 spots in the control cells transfected with siCtrl. In the box plot, center lines show medians; box limits indicate the 25th and 75th percentiles; whiskers extend 1.5 times the interquartile range from the 25th and 75th percentiles. More than 70 cells were analyzed per sample. The two-sided Wilcoxon rank sum test was used for statistical analysis. Middle, protein levels of E2F1 and E2F6 in HeLa cells transfected with siRNAs, siCtrl, siE2F1, or siE2F6, for 48 hours. Right, bar graph generated by quantifying the Western blot. Data are means ± SD, n = 3. Two-sided *t*-test. **h** As for **f**, occupancy of MYC at TUG1 promoter and gene-body region shown by fold-enrichment relative to the chromatin input. Data are mean ± SD, n = 3. Two-sided *t*-test. **i** TUG1 is highly expressed in tumors. TUG1 expression across seven tumors compared to normal tissues in TCGA and the GTEx dataset. TCGA Study Name and the number of samples for tumor and normal tissues are as follows: DLBC: Lymphoid Neoplasm Diffuse Large B-cell Lymphoma, 47, Blood, 337; GBM: Glioblastoma multiforme, 163, Brain, 207; PAAD: Pancreatic adenocarcinoma, 179, Pancreas, 171; THYM: Thymoma, 118, Blood, 339; CHOL: Cholangiocarcinoma, 36, Bile ducts, 9; LGG: Brain Lower Grade Glioma, 518, Brain, 207; LAML: Acute Myeloid Leukemia, 173, Bone Marrow, 70. In the box plot, center lines show medians; box limits indicate the 25th and 75th percentiles; whiskers extend 1.5 times the interquartile range from the 25th and 75th percentiles. One-way ANOVA was used for statistical analysis. TPM: Transcripts per million. **j** The induction of TUG1 expression by treatment with 2 mM HU for 2 h in unsynchronized cancer cell lines. TUG1 expression level normalized by GAPDH was determined using RT-qPCR. Data are mean ± SD, n = 3. Two-sided *t*-test. Source data are provided as a Source Data file.

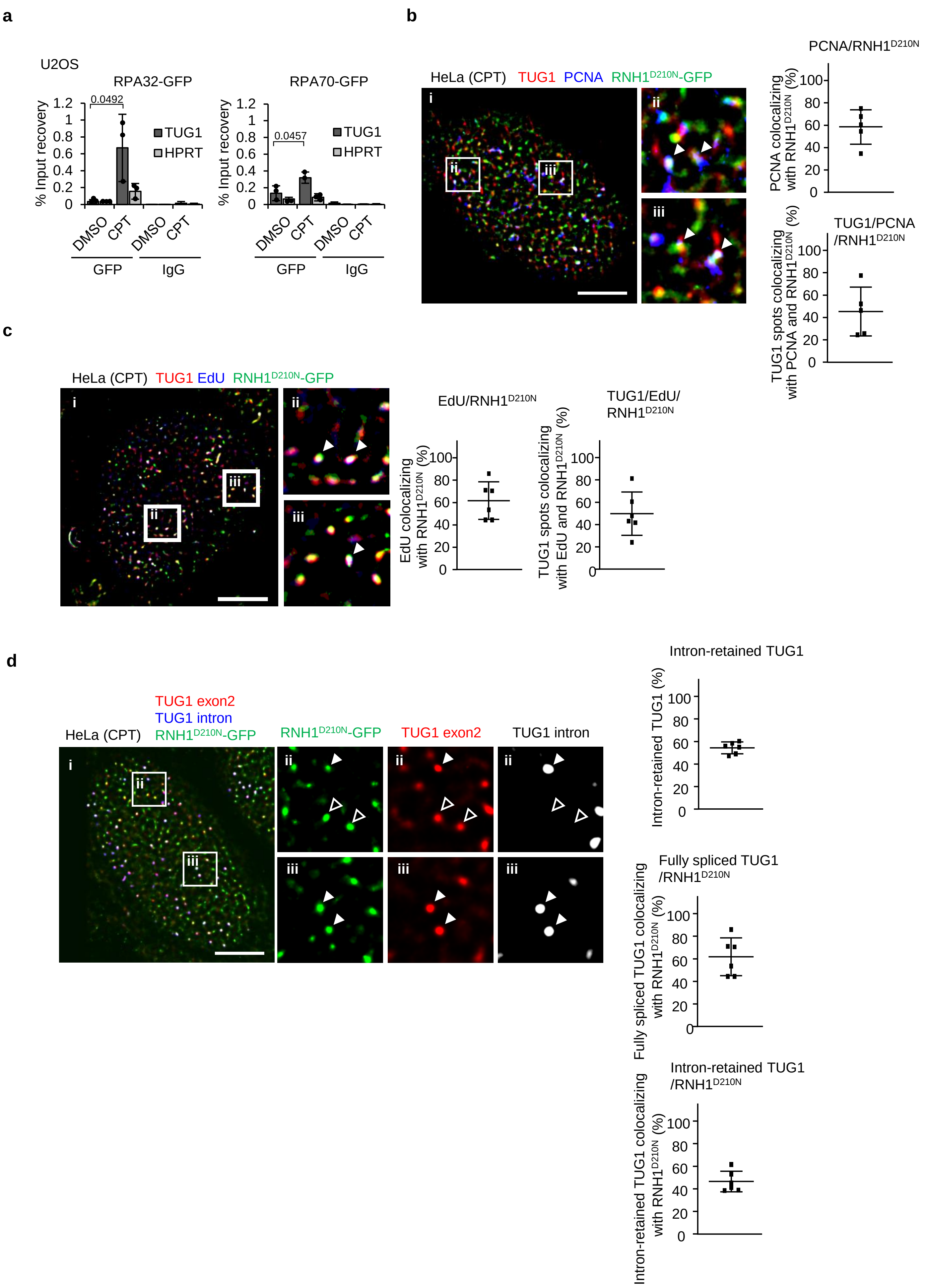

Supplementary Fig. 2. TUG1 colocalizes with RNH1^{D210N}-GFP

Supplementary Fig. 2. TUG1 colocalizes with RNH1^{D210N}-GFP

a RIP assay using GFP antibody indicating the interaction between RPA32 or RPA70 and TUG1 after treatment with 10 μM CPT in U2OS cells expressing RPA32-GFP or RPA70-GFP. HPRT and IgG-bound RNA were taken as negative controls. Data are mean ± SD, n = 3. Two-sided *t*-test. **b** Left, super-resolution images of HeLa cells transfected with catalytically inactive RNase H1 (RNH1^{D210N}-GFP, green) co-stained with TUG1 smFISH (red) and PCNA (blue). Cells were treated with 10 μM CPT for 2 h. (ii, iii) Boxed regions in (i) are shown magnified on the right. Solid arrowheads (white spots) indicate the sites where TUG1, PCNA, and RNH1^{D210N}-GFP are colocalized. Scale bar = 5 μm. Right upper graph indicates the percentage of PCNA spots colocalizing with RNH1^{D210N}-GFP spots relative to the total number of PCNA spots. Right lower graph indicates the percentage of TUG1 spots colocalizing with PCNA and RNH1^{D210N}-GFP spots to the total number of TUG1 spots in each cell, respectively. Cells were treated with 10 μM CPT for 2 h. Median, upper and lower quartile range from 5 independent cells are indicated. Two independent experiments were carried out with similar results (Supplementary Table S2) and a representative image is shown. **c** Left, super-resolution images of HeLa cells transfected with catalytically inactive RNase H1 (RNH1^{D210N}-GFP, green) co-stained with TUG1 smFISH (red) and incorporated EdU (blue). Cells were treated with 10 μM CPT for 2 h and 100 μM EdU for the last 20 mins. (ii, iii) Boxed regions in (i) are shown magnified on the right. Solid arrowheads (white spots) indicate the sites where TUG1, EdU, and RNH1^{D210N}-GFP are co-localized. Scale bar = 5 μm. The percentage of EdU foci colocalizing with RNH1^{D210N}-GFP spots relative to the total number of EdU foci, and the percentage of TUG1 spots colocalizing with EdU foci and RNH1^{D210N}-GFP spots relative to the total number of TUG1 spots in each cell are calculated in the right graphs. Cells were treated with 10 μM CPT for 2 h. Two independent experiments were carried out with similar results (Supplementary Table S2) and a representative image is shown. **d** Left, super-resolution images of HeLa cells transfected with catalytically inactive RNase H1 (RNH1^{D210N}-GFP, green) co-stained with TUG1 smFISH (targeting TUG1 exon 2, red) and TUG1 intron smFISH (targeting intron 1 and intron 2, blue in a, and white in ii and iii). Cells were treated with 10 μM CPT for 2 h. (ii, iii) Boxed regions in (i) are shown magnified on the right. RNH1^{D210N}-GFP spots colocalized with fully spliced (open arrowhead) and intron-retained TUG1 transcripts (solid arrowhead) are indicated. Scale bar = 5 μm. The percentage of TUG1 exon2 spots withTUG1 intron (i.e., intron-retained TUG1) spots relative to the total number of TUG1 spots, the percentage of TUG1 exon2 spots without TUG1 intron spots (i.e., fully spliced TUG1) colocalizing with RNH1^{D210N}-GFP relative to the total number of fully spliced TUG1 spots, and the percentage of intron-retained TUG1 colocalizing with RNH1^{D210N}-GFP relative to the total number of intron-retained TUG1 spots in each cell are calculated in the right graphs. Cells were treated with 10 μM CPT for 2 h. Median, upper and lower quartile range from 6 independent are indicated. Two independent experiments were carried out with similar results (Supplementary Table S2) and a representative image is shown. Source data are provided as a Source Data file.

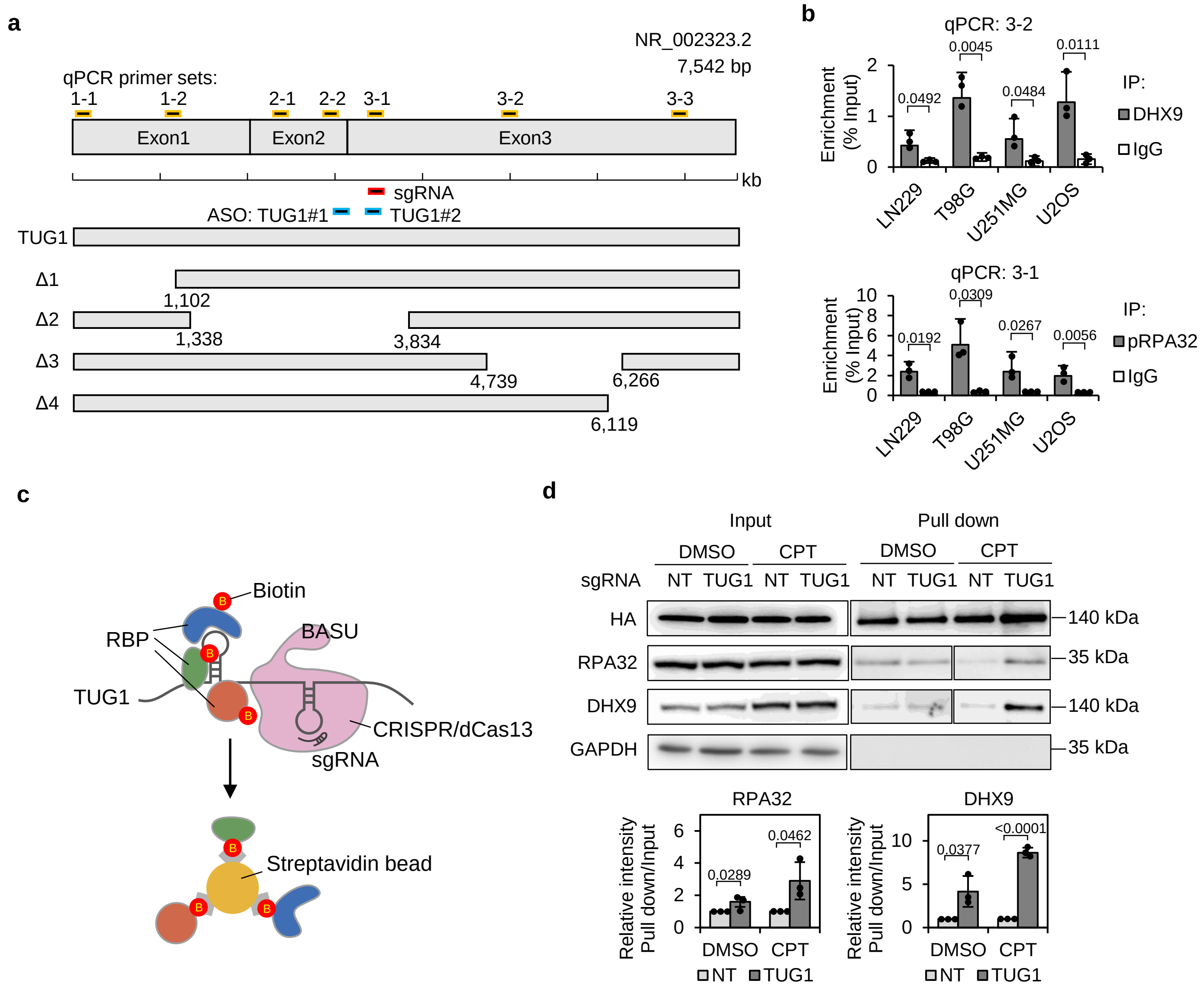

Supplementary Fig. 3. TUG1 interacts with pRPA32 and DHX9

Supplementary Fig. 3. TUG1 interacts with pRPA32 and DHX9

a Schematic diagram of the 7,542 bp of the TUG1 transcript (NR_002323.2) analyzed in our CLIP and RNA pull-down experiments showing the location of the qPCR primer pairs in vertical yellow lines and the structure of the deletion mutants (Δ1-Δ4). The positions of the sgRNA used in CARPID experiments and ASOs (TUG1#1 and TUG1#2) are also indicated. **b** CLIP assay of DHX9 and pRPA32 performed in LN229, T98G, U251MG, and U2OS. Associated TUG1 RNA was quantified by qPCR using primers indicated in Supplementary Fig. S3A. Data are presented as % input, mean ± SD, n = 3. Two-sided *t*-test. **c** Schematic diagram of CARPID. The specific sgRNA-guided CRISPR/dCas13 recognizes the TUG1 single-stranded region. BASU fused to CRISPR/dCas13 adds biotin to adjacent binding proteins (RNA binding proteins, RPB). Streptavidin coated beads are used to purify the binding proteins. **d** Top, TUG1 interacting proteins analyzed by CARPID in HEK293T cells. Western blotting detection of RPA32 and DHX9 in input and streptavidin pulldown samples of control (non-targeting sgRNA: NT) and TUG1 sgRNA (TUG1). CRISPR/dCas13-HA (HA) and GAPDH serves as a positive and negative control, respectively. Bottom, bar graph generated by quantifying the Western blot. Data are normalized to the Input and represent the mean ± SD, n = 3. Two-sided *t*-test. Source data are provided as a Source Data file.

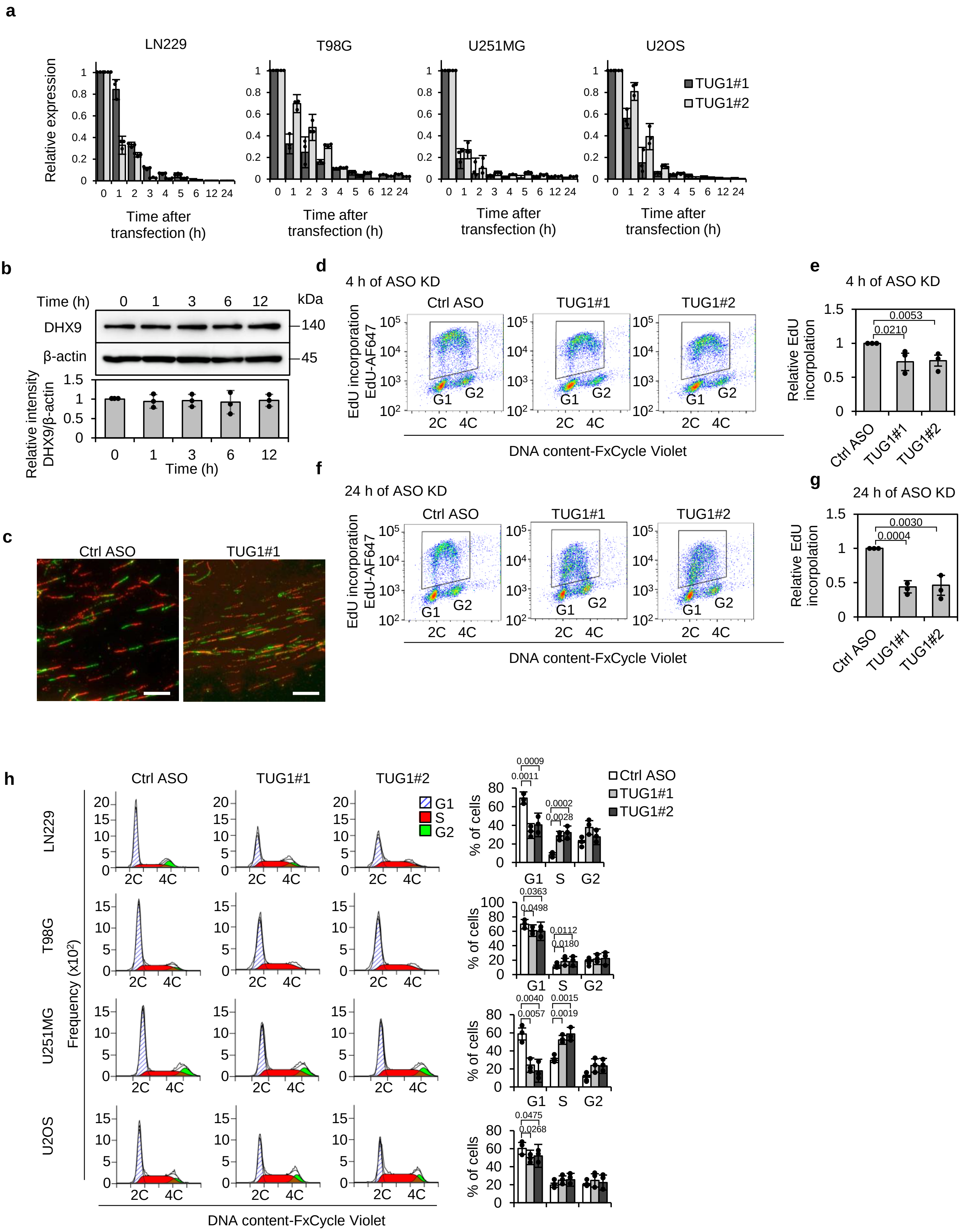

Supplementary Fig. 4. Depletion of TUG1 increases R-loop and RS

Supplementary Fig. 4. Depletion of TUG1 increases R-loop and RS

a KD efficiency was analyzed by RT-qPCR in LN229, T98G, U251MG, and U2OS cells at every hour for 6 h and at 12 h and 24 h after transfection of TUG1 ASO#1 and ASO#2. Expression of TUG1 is normalized to that of GAPDH and mean ± SD, n = 3. **b** Top, TUG1 KD does not affect DHX9 protein expression. DHX9 was detected by Western blotting in HeLa/Fucci2 cells transfected with TUG1 ASO#1 for the indicated time. Bottom, bar graph generated by quantifying the Western blot. Data are normalized to β-actin and represent the mean ± SD, n = 3. Data are not significantly different by two-sided *t*-test. **c** Representative images of spread fibers from HeLa cells transfected with Ctrl ASO or TUG1#1 for 4 h. Cells were first incubated with 25 μM CldU and then with 250 μM iododeoxyuridine IdU for 15 min each. Similar results were obtained in three independent experiments (Supplementary table S4). Scale bar = 5 μm. **d** Representative FCM profiles of EdU incorporation. HeLa cells were treated with Ctrl ASO, TUG1#1 or TUG1#2 for 4 h followed by 1 h of EdU labeling. Cells in the S phase were gated. **e** Relative EdU incorporation in the S phase cells treated with Ctrl ASO, TUG1#1 or TUG1#2 for 4 h. Values are relative to the Ctrl ASO and represented the mean ± SD, n = 3. Two-sided *t*-test. **f** As in **d**, representative FCM profiles of EdU-labeled HeLa cells treated with Ctrl ASO, TUG1#1 or TUG1#2 for 24 h followed by 1 h of EdU incorporation. Cells in the S phase were gated. **g** Relative EdU incorporation in the S phase cells treated with Ctrl ASO, TUG1#1 or TUG1#2 for 24 h. Values are relative to the Ctrl ASO and represented the mean ± SD, n = 3. Two-sided *t*-test. **h** FCM profiles of cells after 24 h of TUG1 KD. Left, cell-cycle distribution of LN229, T98G, U251MG, and U2OS. Right, percentage of cells at each stage of the cell cycle (G1, S, and G2 phases) in four cell lines treated with Ctrl ASO, TUG1#1 or TUG1#2 for 24 h. The cell cycle distribution was analyzed by ModFit LT 5.0 software. Data are mean ± SD, n = 3. Two-sided *t*-test. Source data are provided as a Source Data file.

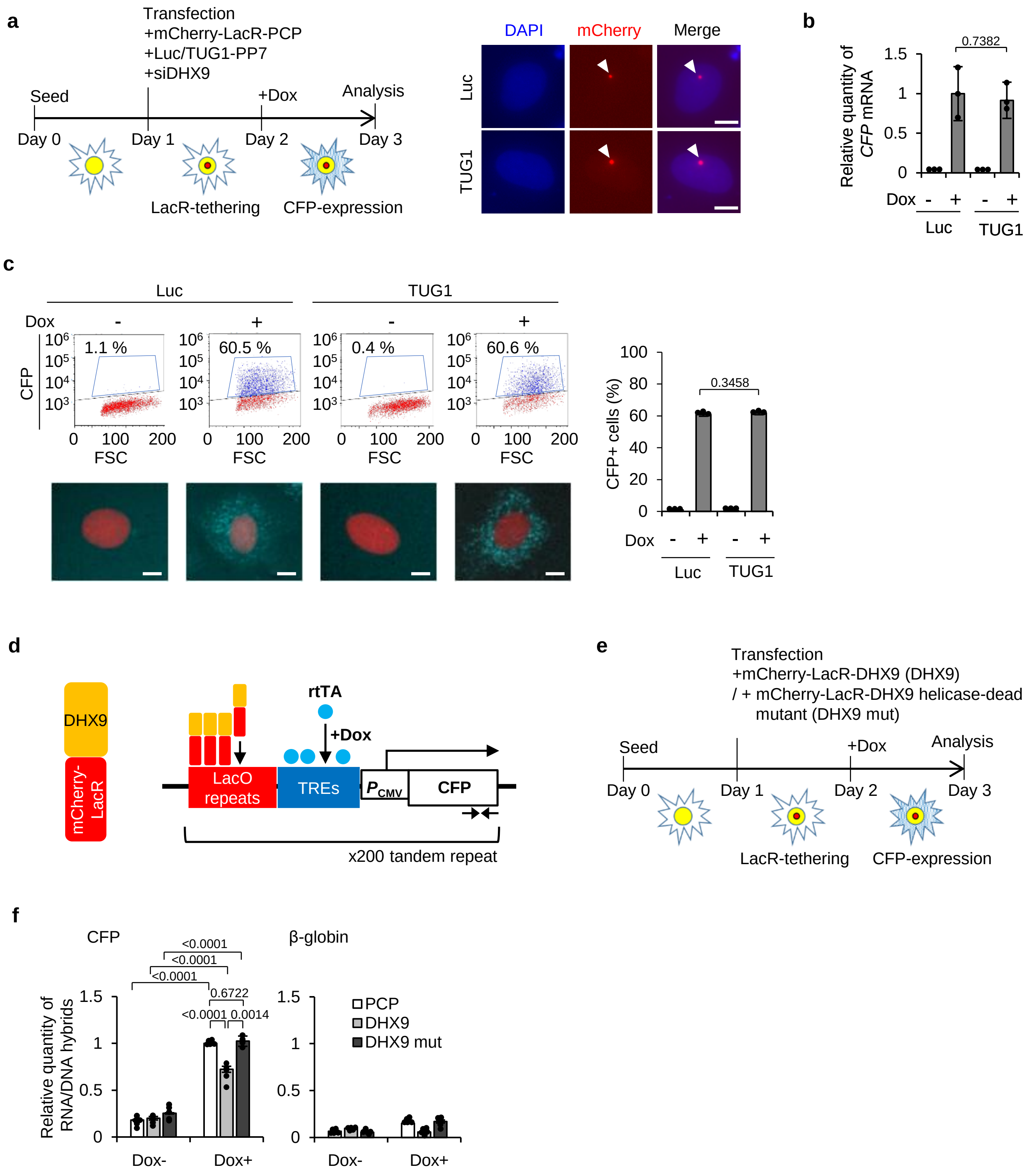

Supplementary Fig. 5. TUG1 tethered to the LacO-repeat resolves R-loops

Supplementary Fig. 5. TUG1 tethered to the LacO-repeat resolves R-loops

a Left, schematic presentation of experimental schedule using U2OS 2-6-3/TA cell lines. Transfection of mCherry-LacR-PCP, Luciferase (Luc) or TUG1-PP7, and siDHX9 was performed one day after seeding (Day 1). Luc or TUG1-PP7 RNAs were assembled on LacO-repeat loci through the LacO-LacR system combined with PP7-PCP. The LacO-repeat loci is visualized as mCherry foci indicated by the red dot in the nucleus (yellow circles). To analyze the accumulation of RNA/DNA hybrids, the transfected U2OS 2-6-3/TA cells were treated with Dox to induce CFP expression (blue dots in cytoplasm) for 24 h (from Day 2 to Day 3). Right, representative images of mCherry foci-formation on LacO-repeat region. Scale bar = 10 µm. **b** Expression of CFP is not affected by tethering of TUG1-PP7. Relative expression of CFP to GAPDH at Day 3 is shown. Values are relative to expression of CFP in cells expressing Luc-PP7 under Dox induction. Data are mean ± SD, n = 3. Two-sided *t*-test. **c** Top left, cells were subjected to FCM analysis for CFP-expression on day 3. Percentage of CFP-positive cells in mCherry-positive cells is indicated. Bottom left, representative images of cells analyzed by FCM. Scale bar = 10 µm. Right, bar graph shows percentage of CFP-positive cells. Data are mean ± SD, n = 3. Two-sided *t*-test. **d** Schematic presentation of tethering of DHX9 on LacO-repeat regions by the LacO-LacR system in U2OS 2-6-3/TA cells combined with Dox-inducible transcriptional activation. Arrows indicate primer positions for DRIP-qPCR at CFP transcribed regions. **e** Schematic presentation of experimental schedule using U2OS 2-6-3/TA cell lines. Transfection of mCherry-LacR-DHX9 (DHX9) or mCherry-LacR-DHX9 helicase-dead mutant (DHX9 mut; D511A and E512A) was performed one day after seeding (Day 1). DHX9 was tethered on LacO-repeat loci through the LacO-LacR system. The LacO-repeat loci are visualized as mCherry foci indicated by the red dot in the nucleus (yellow circles). To analyze the accumulation of RNA/DNA hybrids, the transfected U2OS 2-6-3/TA cells were treated with 3 µg/ml Dox to induce CFP expression (blue dots in the cytoplasm) for 24 h (from Day 2 to Day 3). **f** Quantification of RNA/DNA hybrids accumulated in transcribed CFP gene by DRIP-qPCR assay. U2OS 2-6-3/TA cells expressing either mCherry-LacR-DHX9 (DHX9) or mCherry-LacR-DHX9 helicase-dead mutant (DHX9 mut; D511A and E512A) were cultured with (Dox+) or without Dox (Dox-) for 24 h. mCherry-LacR-PCP (PCP) was used as a control. Values are relative to RNA/DNA hybrids accumulated in transcribed CFP gene in cells expressing PCP and cultured with Dox. Data are mean ± SE, n = 5. Two-sided *t*-test. Source data are provided as a Source Data file.

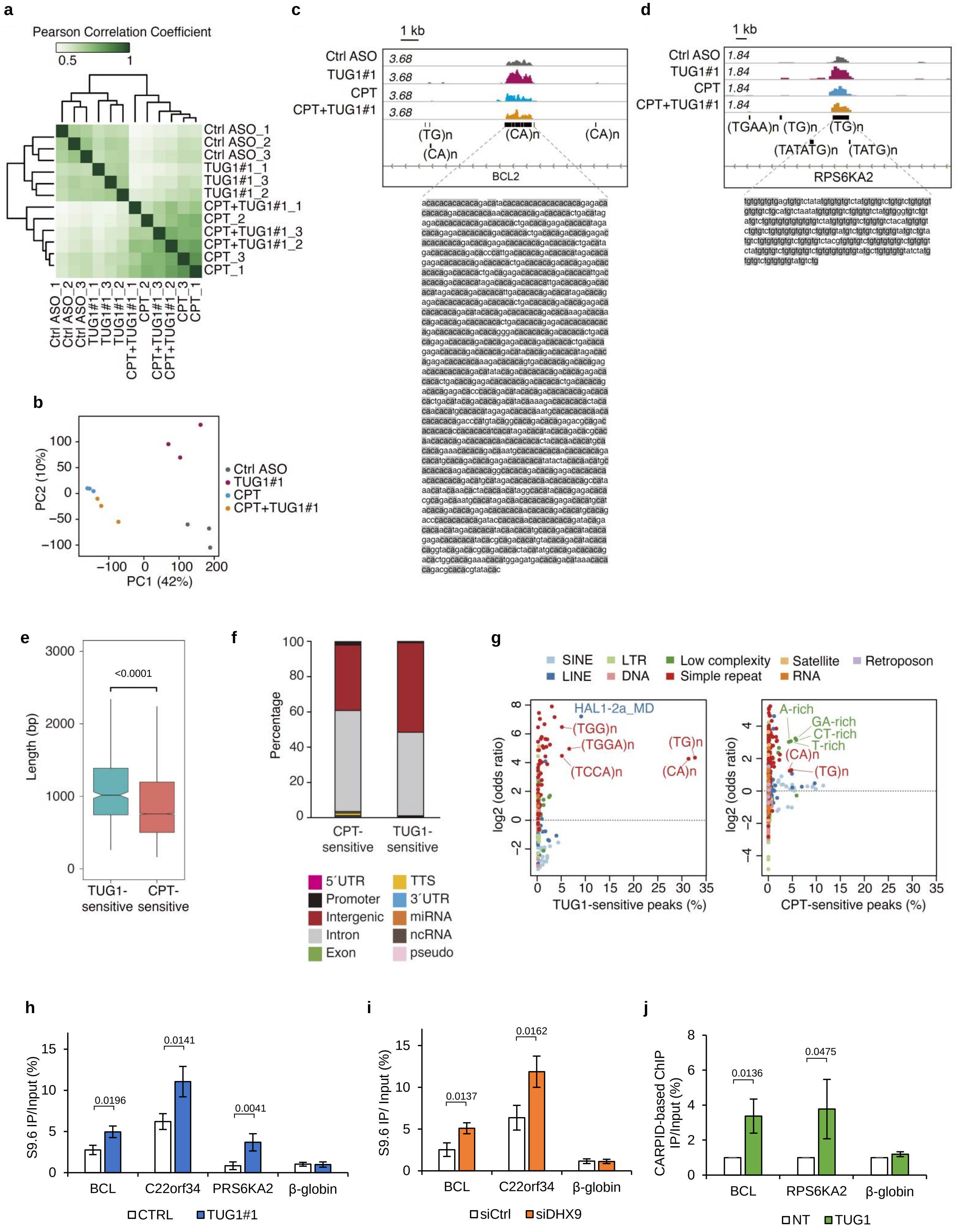

Supplementary Fig. 6. DRIP-seq analysis in TUG1 depleted cells

Supplementary Fig. 6. DRIP-seq analysis in TUG1-depleted cells

a Pearson correlation heatmap of DRIP-seq data across all triplicate samples. The color bar represents the correlation coefficient (r) with the dark green side being positive correlations and the white side the negative correlations. **b** Principal Component Analysis of DRIP-seq. **c** Snapshots of representative loci of DRIP-seq peaks enhanced by TUG1 KD in chr18:60,865,264-60,867,074 (BCL2). (CA)n repeat containing sequence is indicated below with CA dinucleotide colored gray. **d** Snapshots of representative loci of DRIP-seq peaks enhanced by TUG1 KD in chr6:167,237,925-167,238,369 (RPS6KA2). (TG)n repeat containing sequence is indicated below with TG dinucleotide colored gray. **e** Peak length distribution of TUG1-sensitive (n=528) and CPT-sensitive peaks (n=19,191). In the box plot, center lines show medians; box limits indicate the 25th and 75th percentiles; whiskers extend 1.5 times the interquartile range from the 25th and 75th percentiles. Mann-Whitney U-test. **f** Genomic annotation of peaks differentially altered by TUG1 KD or CPT treatment, defined by homer. Data are also summarized in Supplementary Table S5. **g** Additional simple repeat enrichment analysis in TUG1-sensitive (left) or CPT-sensitive (right) peaks. The x-axis shows the fraction of TUG1- or CPT-sensitive peaks overlapping with the indicated repeat types and sequences in RepeatMasker. The y- axis shows Odds Ratios for the occurrence of the indicated repeat types and sequences above background. Odds Ratios were estimated by comparison with GC%-matched background regions for TUG1- or CPT-sensitive peaks. **h** DRIP-qPCR validation regions called as TUG1-sensitive peaks (BCL2, C22orf34, and PRS6KA2 loci). β-globin locus is used as a control. Genomic DNA were derived from HeLa cells treated with Ctrl ASO or TUG1#1. Data are mean ± SD, n = 3. Two-sided *t*-test. **i** DRIP-qPCR of BCL2 and C22orf34 loci in genomic DNA derived from HeLa cells treated with siCtrl or siDHX9. Data are mean ± SD, n = 3. Two-sided *t*-test. **j** CARPID-based ChIP-qPCR in HEK293T cells. TUG1-proximity proteins biotinylated by CRISPR-assisted system were crosslinked with chromatin and pulled down with streptavidin beads. Associated DNA was quantified by qPCR. Data are presented as % input, mean ± SD, n = 3. Two-sided *t*-test. Source data are provided as a Source Data file.

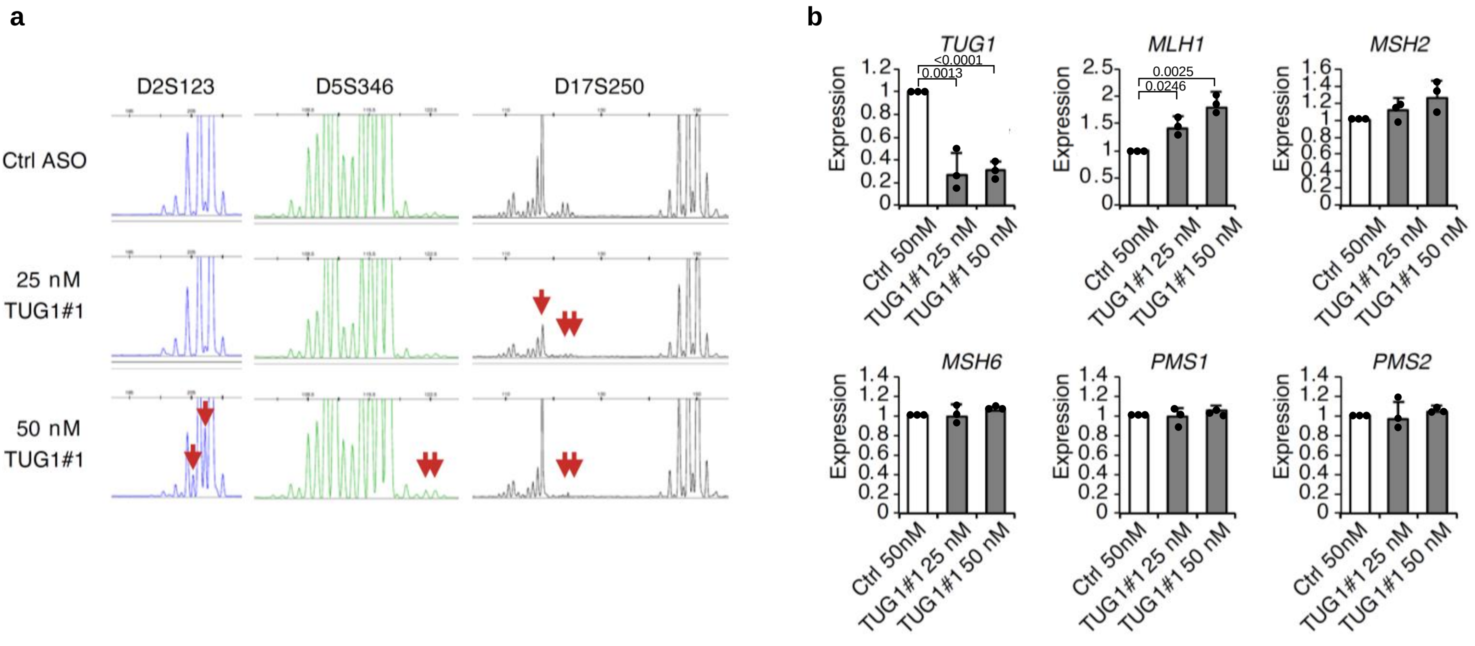

Supplementary Fig. 7. TUG1 enhances MSI in MMR-proficient cells

a Representative images of MSI in T98G (D5S346) and LN229 (D2S123 and D17S250) cells. The red arrows indicate aberrant microsatellite repeat elements, distinct in molecular weight and thus mobility on capillary electrophoretograms from those in the Ctrl ASO treated cells. **b** Expression of MMR genes MLH1, MSH2, MSH6, PMS1 and PMS2 is not downregulated by TUG1 KD at the indicated concentrations for two weeks. Expression levels of MMR genes are normalized to those of GAPDH. The y-axis indicates relative expression level compared to Ctrl ASO-treated cells. Data are mean ± SD, n = 3. Two-sided *t*-test. Source data are provided as a Source Data file.

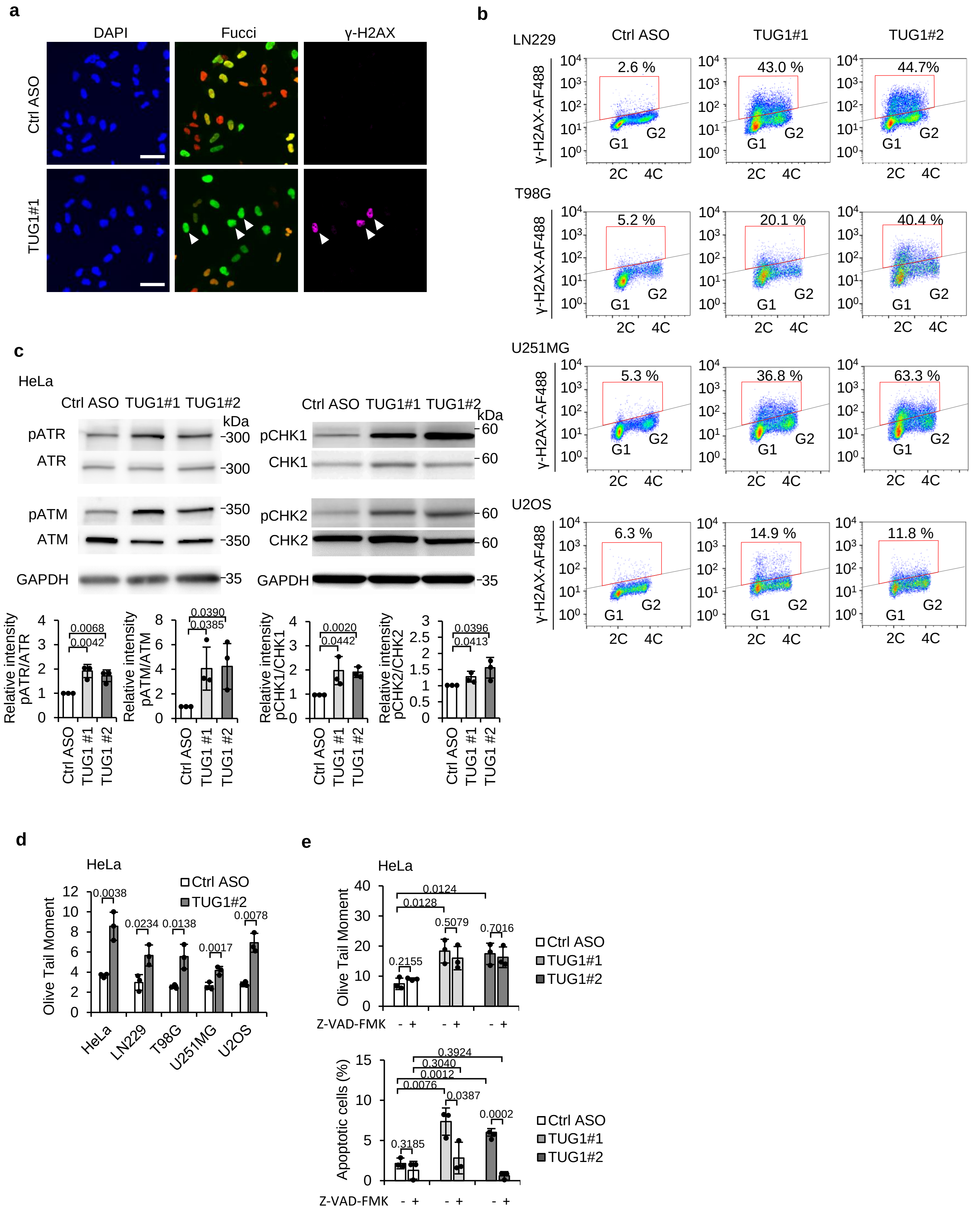

Supplementary Fig. 8. TUG1 depletion causes DNA damage in cancer cells

Supplementary Fig. 8. TUG1 depletion causes DNA damage in cancer cells

a HeLa/Fucci2 cells transfected with Ctrl ASO, or TUG1#1 for 24 h were immunostained with anti-γ-H2AX antibody. Fluorescent ubiquitination-based cell cycle indicator (Fucci) visualized cell cycle progression; G1 cells and S/G2 cells are shown in red and green fluorescence, respectively. γ-H2AX-positive cells are indicated by white arrowheads. Scale bar = 50 µm. Three independent experiments were carried out with similar results and a representative image is shown. **b** Representative FCM profiles of four cell lines after 24 h of TUG1 KD. γ-H2AX-positive cells against DNA content are shown. **c** Top, western blotting of HeLa/Fucci2 cells transfected with Ctrl or TUG1 ASO for 24 h. Anti-phospho (Thr1989) ATR (p-ATR), anti-ATR, Anti-phospho (Ser1981) ATM (p-ATM), anti-ATM, Anti-phospho (Ser345), CHK1 (p-Chk1), anti-CHK1, anti-phospho (Thr68) CHK2 (p-CHK2), and anti-CHK2 antibodies were used. Bottom, bar graphs generated by quantifying the Western blot. The relative intensities of pATR normalized by ATR, pATM normalized by ATM, pCHK1 normalized by CHK1, and pCHK2 normalized by CHK2 were indicated. Data are means ± SD, n = 3. Two-sided *t*-test. **d** Detection of DSBs by neutral comet assays. Bar graph shows quantification of Olive tail moment in Ctrl ASO or TUG1#2-transfected HeLa cells. 100 cells per group were examined. Dots indicate mean value of the 100 cells. Error bars represent mean ± SD. n=3, Two-sided *t*-test. **e** Inhibition of apoptosis by caspase inhibitor Z-VAD-FMK. Top, quantification of Olive tail moment in Ctrl ASO, TUG1#1, or TUG1#2-transfected HeLa cells with or without 20 µM Z-VAD-FMK. 100 cells per group were examined. Dots indicate mean value of the 100 cells in three individual experiments. Error bars represent mean ± SD. Two-sided *t*-test. Bottom, percentages of cells with hedgehog comets represent cells in early apoptosis. 100 cells per group were examined. Dots indicate mean value of the 100 cells in three individual experiments. Error bars represent mean ± SD. n = 3. Two-sided *t*-test. Source data are provided as a Source Data file.

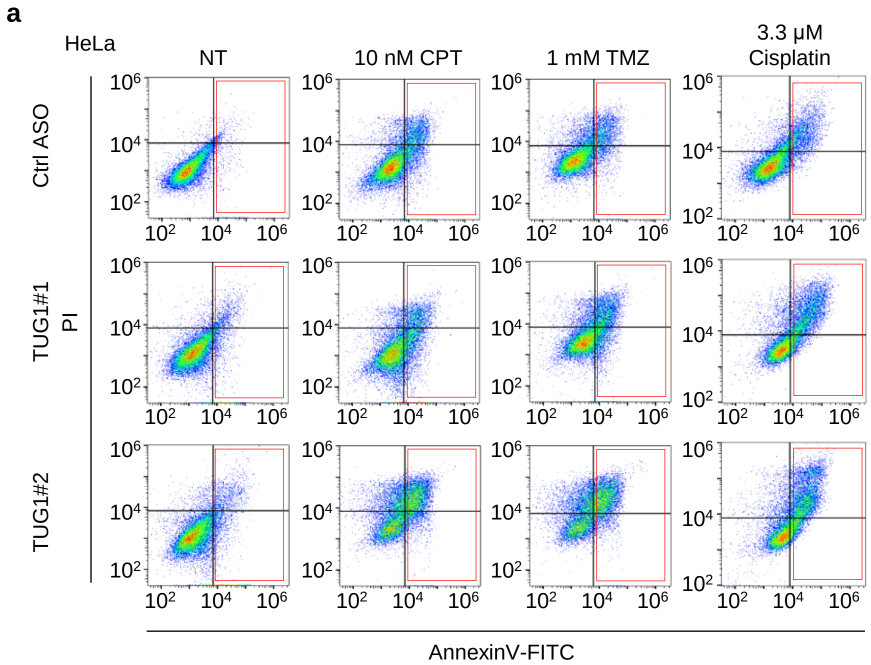

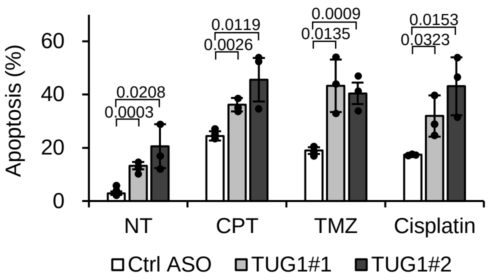

	Ctrl ASO	TUG1 ASO#1	
	IC50	IC50	CI
CPT	22.4 nM	10.3 nM	0.7
TMZ	1,765 μM	1,171 μM	0.8
Cisplatin	7.5 μM	5.3 μM	0.7

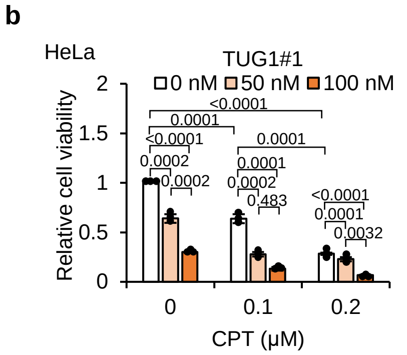

CPT (μM)	0.1		0.2	
TUG1#1 (nM)	50	100	50	100
CI	0.73	0.53	0.81	0.35

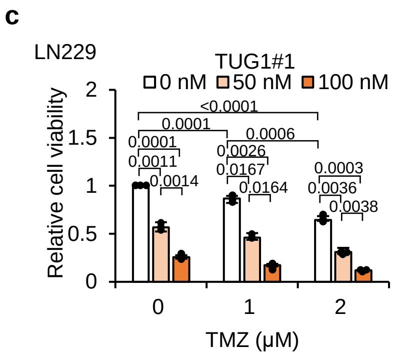

TMZ (μM)	1.0		2.0	
TUG1#1 (nM)	50	100	50	100
CI	0.84	0.67	0.50	0.53

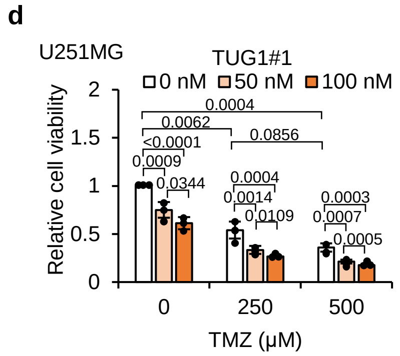

TMZ (μM)	250		500	
TUG1#1 (nM)	50	100	50	100
CI	0.38	0.33	0.31	0.25

Supplementary Fig. 9. Inhibition of TUG1 enhances the chemosensitivity
a Left, Annexin V/PI staining of HeLa cells transfected with Ctrl ASO or TUG1 ASO for 48 h were exposed to 10 nM CPT, 1 mM TMZ, or 3.3 μM cisplatin for the last 24 h. Top right, percentages of Annexin V-positive cells. Error bars represent mean ± SD. n = 3. Two-way ANOVA and Tukey's multiple comparison tests. Bottom right, values of 50% inhibitory concentration (IC50) and combination index (CI) in HeLa cells. CI was calculated using CompuSyn software. **b** Top, influence of TUG1 ASO on cell viability in combination with CPT. HeLa cells were transfected with 50 nM or 100 nM TUG1#1 in the presence of CPT at the indicated concentrations for 48 h. The obtained data are normalized to the non-treated control and shown as relative cell viability. Error bars represent mean ± SD. n = 3. Two-sided *t*-test. Bottom, values for the combination index (CI) in HeLa cells treated with TUG1 ASO in combination with CPT were calculated. **c, d** Top, as for **a**, influence of TUG1 ASO on cell viability in combination with TMZ. LN229 cells (**c**) or U251MG cells (**d**) were transfected with 50 nM or 100 nM TUG1#1 in the presence of TMZ at the indicated concentrations for 48 h. The obtained data are normalized to the non-treated control and shown as relative cell viability. Bottom, values of the combination index (CI) in LN229 (**c**) or U251MG (**d**) treated with TUG1 ASO in combination with TMZ. Error bars represent mean ± SD. n = 3. Two-sided *t*-test. Source data are provided as a Source Data file.

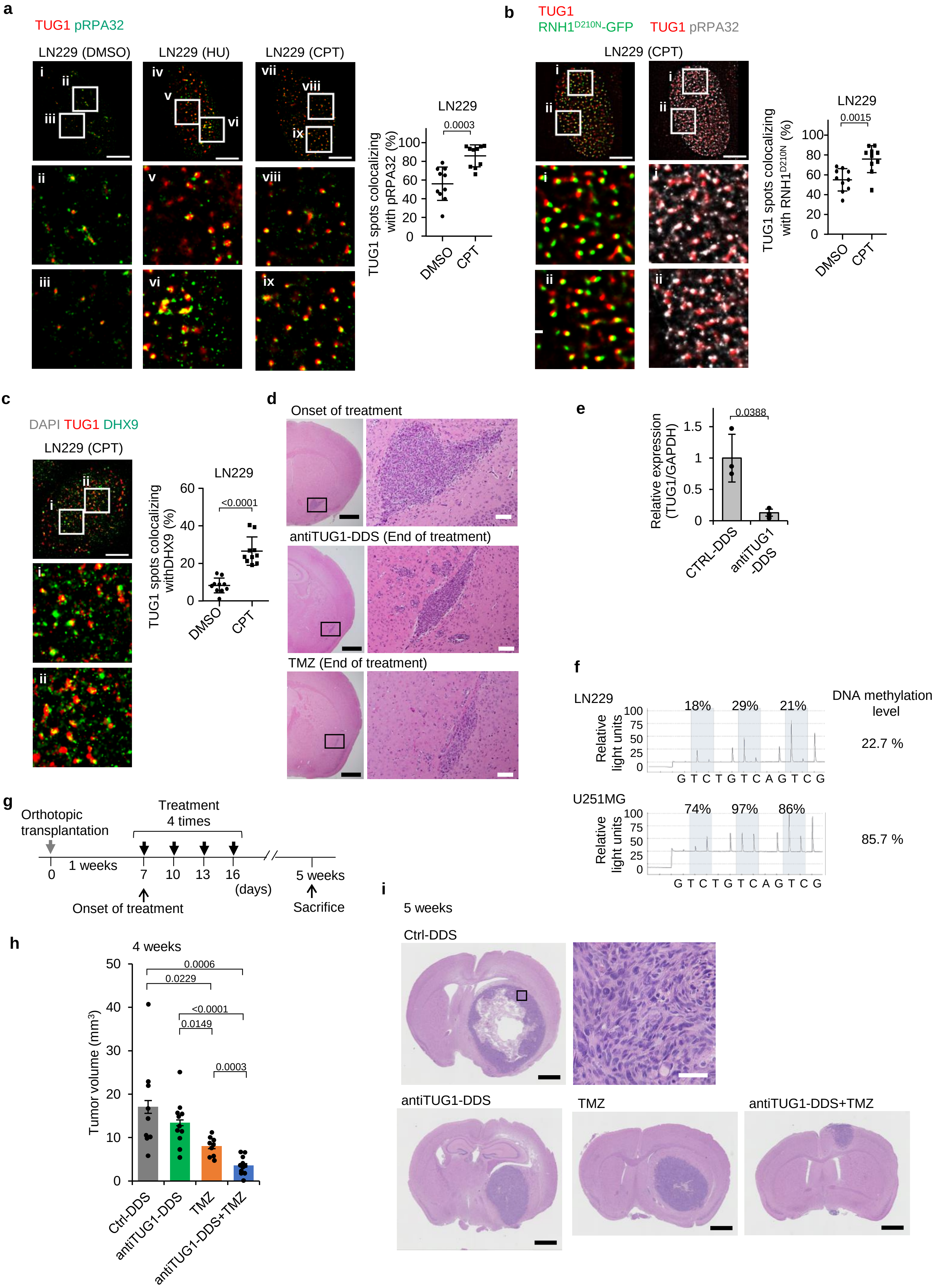

Supplementary Fig. 10. Combination therapy with antiTUG1-DDS and TMZ suppresses tumor g rowth in glioblastoma xenograft mouse model

Supplementary Fig. 10. Combination therapy with antiTUG1-DDS and TMZ suppresses tumor growth in a LN229 glioblastoma xenograft mouse model

a Right, super-resolution images of LN229 cells co-stained with pRPA32 (green) and TUG1 smFISH (red). (i-iii) LN229 cell treated with DMSO (untreated control). (iv-vi) LN229 cell treated with 2 mM HU for 2 h. (vii-ix) LN229 cell treated with 10 µM CPT for 2 h. Bottom panels are magnified regions where TUG1 and pRPA32 colocalize in the top panels. Three independent experiments were carried out with similar results (Supplementary Table S2) and representative images are shown. Scale bar = 5 µm. Left, the percentage of TUG1 spots colocalizing with pRPA32 relative to total number of TUG1 spots in each cell. Cells were treated with DMSO or 10 µM CPT for 2 h. Median, upper and lower quartile range from 10 independent cells are indicated. Two-sided *t*-test. **b** Left, Super-resolution images of LN229 cell transfected with catalytically inactive RNase H1 ($RNH1^{D210N}$-GFP, green) co-stained with pRPA32 (white) and TUG1 smFISH (red). Cell was treated with 10 µM CPT for 2 h. Magnified regions in the top panel were shown in middle (i) and bottom (ii) panels. Three independent experiments were carried out with similar results (Supplementary Table S2) and a representative image is shown. Scale bar = 5 µm. Right, the percentage of TUG1 spots colocalizing with $RNH1^{D210N}$-GFP relative to total number of TUG1 spots in each cell. Median, upper and lower quartile range from 10 independent cells are indicated. Two-sided *t*-test. **c** Super-resolution images of LN229 cell co-stained with DHX9 (green) and TUG1 smFISH (red) treated with 10 µM CPT for 2 h. Magnified regions in the top panel were shown in middle (i) and bottom (ii) panels. Three independent experiments were carried out with similar results (Supplementary Table S2), and representative images are shown. Scale bar = 5 µm. Right, the percentage of TUG1 spots colocalizing with DHX9 relative to total number of TUG1 spots in each cell. Median, upper and lower quartile range from 10 cells are indicated. Two-sided *t*-test. **d** Representative HE-stained brain sections. Black and white scale bars are 10 mm and 20 µm, respectively. Boxed regions in the left panels are magnified in the right panels. **e** RNA expression levels of TUG1 in the tumor cells of mouse xenografts. Expression levels of TUG1 are normalized to those of GAPDH. The y-axis indicates relative expression level compared to CTRL-DDS. CTRL-DDS (n = 3), antiTUG1-DDS (n = 3), mean ± SD. Two-sided *t*-test. **f** Representative pyrograms by pyrosequencing analyses of *MGMT* promoter for LN229 (top) and U251 (bottom); shadow squares highlight each CpG site analyzed by pyrosequencing with the corresponding percentage of methylation calculated by the software. The experiments were conducted in duplicate and average value was calculated. **g** Schematic diagram showing the treatment protocol for xenograft mouse models of U251MG. **h** Tumor volumes at 4 weeks after transplantation. CTRL-DDS (n = 10), antiTUG1-DDS (n = 11), TMZ (n = 9), antiTUG1-DDS+TMZ (n = 11). Error bars indicate SE. Two-sided *t*-test. **i** Representative HE-stained brain sections 5 weeks after transplantation. Boxed region in Ctrl-DDS panel is shown magnified in the right panel. Black and white scale bars are 10 mm and 10 µm, respectively. Source data are provided as a Source Data file.

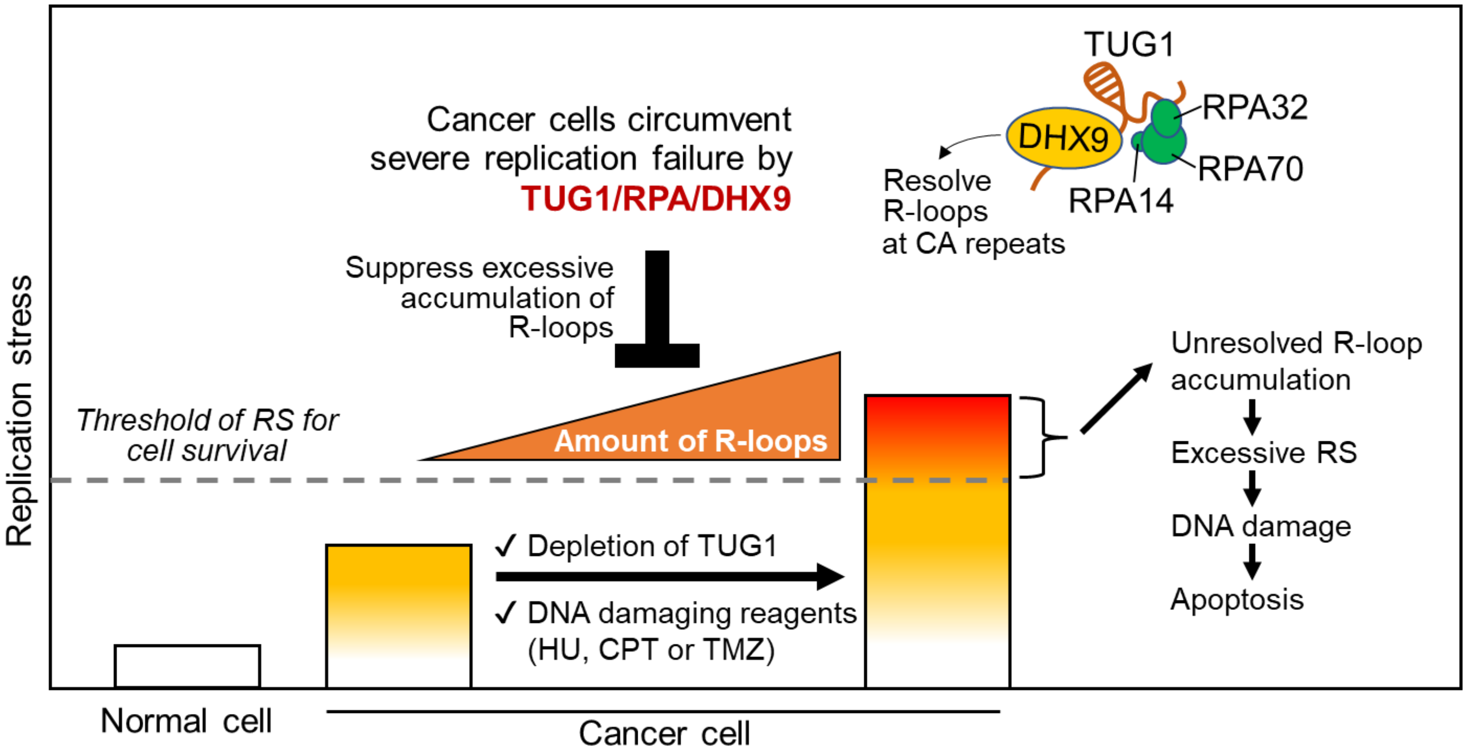

Supplementary Fig. 11. Cancer cells circumvent severe replication failure by TUG1/RPA/DHX9
Schema showing the proposed mechanisms of TUG1-mediated R-loop resolution under RS. Depletion of TUG1 fails to resolve accumulated R-loops and results in DNA damage and apoptosis.

a

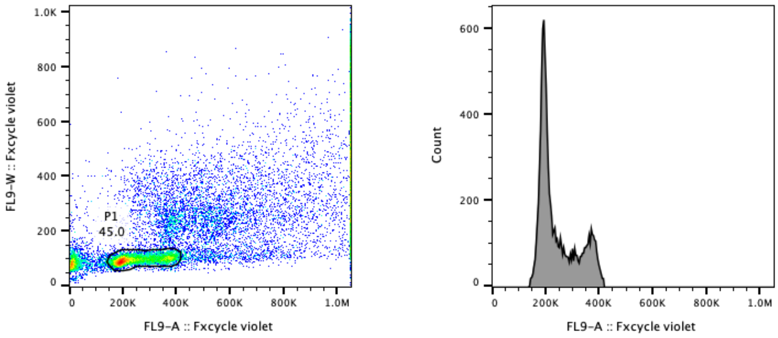

Ungated #Events	Ungated %Total	P1 #Events	P1 %Total
21684	100	9749	45.0

b

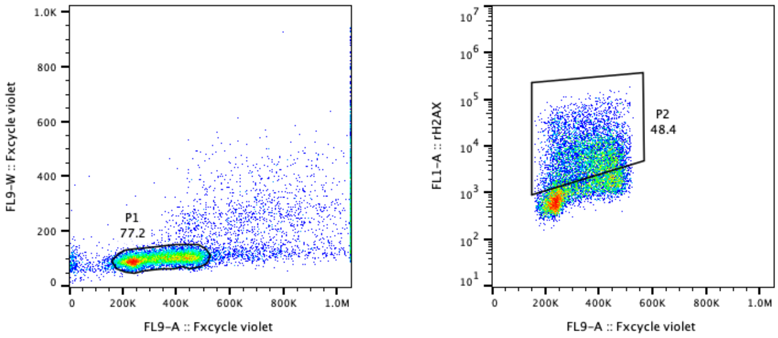

Ungated #Events	Ungated %Total	P1 #Events	P1 %Total	P2 #Events	P2 %Parent
15052	100	11616	77.2	5623	48.4

c

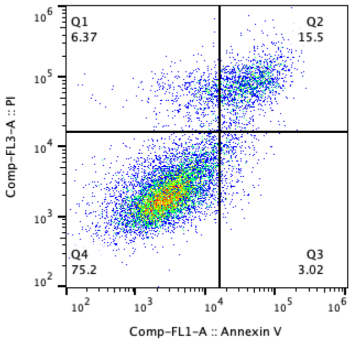

Ungated #Events	Ungated %Total	Q1 #Events	Q1 %Total	Q2 #Events	Q2 %Total	Q3 #Events	Q3 %Total	Q4 #Events	Q4 %Total
10310	100	657	6.37	1594	15.5	311	3.02	7748	75.2

d

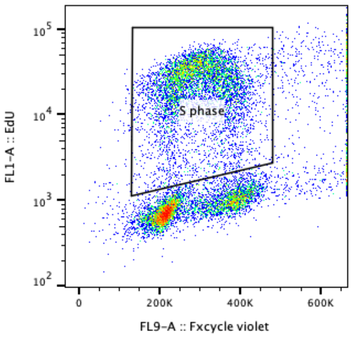

Ungated #Events	Ungated %Total	S phase #Events	S phase %Total
13319	100	6471	48.6

e

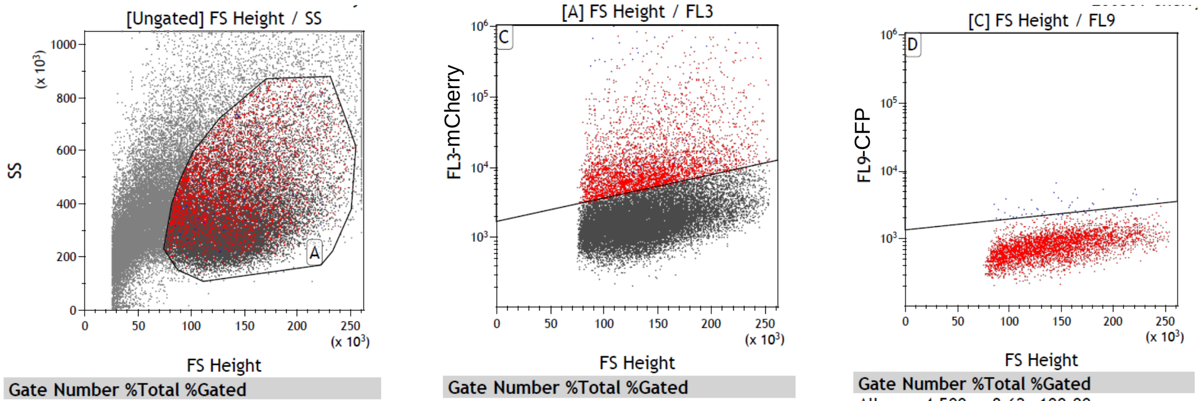

Gate Number		%Total	%Gated
All	52,180	100.00	100.00
A	27,462	52.63	52.63

Gate Number		%Total	%Gated
All	27,462	52.63	100.00
C	4,500	8.62	16.39

Gate Number		%Total	%Gated
All	4,500	8.62	100.00
D	51	0.10	1.13

Supplementary Fig. 12. FCM gating strategy
a Cell cycle analysis for Fig. 4d and Supplementary Fig. S4h. **b** γ-H2AX and DNA content analysis fo r Fig. 6b, Fig. 6F, and Supplementary Fig. 8b. **c** Apoptosis analysis for Fig. 6e, Fig. 6f, Fig. 7b, and Supplementary Fig. 9a. **d** EdU incorporation analysis for Supplementary Fig. 4d and Fig. 4f. **e** Analysis of mCherry and CFP positive cells for Supplementary Fig. 5c.

Supplementary Table 1. Summary of super resolution microscopic analysis

SD; standard deviation. *P* -values were calculated by *t* -test.

						% of TUG1 colocalized with **pRPA32**		
	Cell line	**Protein**	Experiment	Treatment	no. of cells examined	median	SD	*P* -value
Fig. 2A, B	HeLa	pRPA32	#1	DMSO	10	31.9	9.3	4.22147E-07
				CPT	10	66.0	14.3	
			#2	DMSO	5	21.4	6.4	0.00064
				CPT	4	61.5	26.0	
			#3	DMSO	4	22.3	6.8	0.00004
				CPT	5	69.1	12.1	
	U2OS	pRPA32	#1	DMSO	10	18.2	4.6	7.17336E-08
				CPT	10	57.2	15.4	
			#2	DMSO	4	20.3	7.3	0.0012
				CPT	4	46.6	12.3	
			#3	DMSO	4	19.3	4.5	0.0023
				CPT	4	56.0	22.2	
	U2OS	pRPA32	#1	DMSO	4	13.8	1.9	0.015
				CPT	4	33.8	22.7	
			#2	DMSO	4	4.2	0.7	0.02
				CPT	4	33.0	32.7	
			#3	DMSO	4	12.7	10.9	0.009
				CPT	4	54.4	25.6	

						% of TUG1 colocalized with **pRPA32**	
	Cell line	**Protein**	Experiment	Treatment	no. of cells examined	median	SD
Fig. 2C	HeLa	γ-H2AX	#1	CPT	4	1.8	1.5
			#2	CPT	5	3.5	0.9
			#3	CPT	4	3.4	2.0

						% of TUG1 colocalized with **$RNH1^{D210N}$-GFP**		
	Cell line	**Protein**	Experiment	Treatment	no. of cells examined	median	SD	*P* -value
Fig. 2E	HeLa	$RNH1^{D210N}$-GFP	#1	DMSO	10	21.1	5.9	1.4782E-06
				CPT	10	62.2	20.0	
			#2	DMSO	4	28.0	8.2	0.0013
				CPT	4	52.7	8.0	
			#3	DMSO	4	13.9	6.5	0.0056
				CPT	6	51.8	24.3	

						% of TUG1 colocalized with **DHX9**		
	Cell line	**Protein**	Experiment	Treatment	no. of cells examined	median	SD	*P* -value
Fig. 3C	HeLa	DHX9	#1	DMSO	10	5.4	6.4	1.70E-07
				CPT	10	24.0	6.1	
			#2	DMSO	4	2.7	6.8	0.00025
				CPT	4	27.7	5.6	
			#3	DMSO	4	6.1	3.8	0.02
				CPT	4	23.1	15.0	

						% of TUG1 colocalized with **PCNA** and **$RNH1^{D210N}$-GFP**		% of TUG1 colocalized with **PCNA**		% of **PCNA** colocalized with **$RNH1^{D210N}$-GFP**	
	Cell line	**Protein**	Experiment	Treatment	no. of cells examined	median	SD	median	SD	median	SD
Supplementary Fig. 2B	HeLa	PCNA and $RNH1^{D210N}$-GFP	#1	CPT	5	45.3	21.8	58.6	7.7	58.5	15.4
			#2	CPT	4	41.2	14.4	68.9	23.8	55.7	29.1

						% of TUG1 colocalized with **EdU** and **$RNH1^{D210N}$-GFP**		% of TUG1 colocalized with **EdU**		% of TUG1 colocalized with **$RNH1^{D210N}$-GFP**	
	Cell line	**Protein**	Experiment	Treatment	no. of cells examined	median	SD	median	SD	median	SD
Supplementary Fig. 2C	HeLa	EdU labeling and $RNH1^{D210N}$-GFP	#1	CPT	6	45.4	19.3	58.0	20.9	62.2	16.8
			#2	CPT	4	34.7	20.6	66.9	16.2	42.8	22.0

						% of **fully spliced TUG1** colocalized with **$RNH1^{D210N}$-GFP**		% of **intron-retained TUG1** colocalized with **$RNH1^{D210N}$-GFP**		% of **intron-retained TUG1**	
	Cell line	**Protein**	Experiment	Treatment	no. of cells examined	median	SD	median	SD	median	SD
Supplementary Fig. 2D	HeLa	$RNH1^{D210N}$-GFP	#1	CPT	6	50.4	3.7	46.5	8.4	54.4	4.8
			#2	CPT	5	65.2	10.9	52.6	9.6	61.3	8.3

						% of TUG1 colocalized with **pRPA32**		
	Cell line	**Protein**	Experiment	Treatment	no. of cells examined	median	SD	*P* -value
Supplementary Fig. 10A, B	LN229	pRPA32	#1	DMSO	10	57.6	17.9	0.00019
				CPT	10	92.8	11.7	
			#2	DMSO	4	30.2	24.2	0.05
				CPT	4	78.6	16.0	
			#3	DMSO	4	45.2	11.8	0.012
				CPT	4	74.2	11.5	

	Cell line	**Protein**	Experiment	Treatment	no. of cells examined	% of TUG1 colocalized with **RNH1^{D210N}-GFP** median	SD	*P*-value
Supplementary Fig. 10C	LN229	RNH1^{D210N}-GFP	#1	DMSO	10	55.2	11.2	0.0016
				CPT	10	80.7	13.6	
			#2	DMSO	4	56.3	11.2	0.02
				CPT	4	78.1	13.6	
			#3	DMSO	4	60.5	5.7	0.029
				CPT	4	81.4	8.7	

	Cell line	**Protein**	Experiment	Treatment	no. of cells examined	% of TUG1 colocalized with **DHX9** median	SD	*P*-value
Supplementary Fig. 10D	LN229	DHX9	#1	DMSO	11	11.5	6.7	1.75E-06
				CPT	10	24.0	6.4	
			#2	DMSO	4	13.3	6.1	0.027
				CPT	4	26.1	7.8	
			#3	DMSO	4	10.4	3.5	0.064
				CPT	4	17.7	8.0	

Supplementary Table 2. Top proteins identified by mass spectrometry from excised bands around 140 kDa					
**Protein**	**Gene**	**Accession Number**	**Molecular Weight**	**Exponentially modified protein abundance index (emPAI)[1] values**	
				**sense TUG1 RNA**	**antisense TUG1 RNA**
Bovine Albumin (experimentaly contaminant peptides)			69 kDa	69	63
Myosin-1	MYH1	P12882	223 kDa	19	27
Trypsin (experimentaly contaminant peptides)			24 kDa	17	16
Cluster of Myosin-7	MYH7	P12883	223 kDa	14	11
ATP-dependent RNA helicase A	DHX9	Q08211	141 kDa	9	1
Keratin, type II cytoskeletal 1	KRT1	P04264	66 kDa	8	12
Actin, cytoplasmic 1	ACTB	P60709	42 kDa	7	6
Tropomyosin 1 (Alpha), isoform CRA_o	TPM1	A0A0S2Z4G6	33 kDa	6	4
Keratin, type I cytoskeletal 10	KRT10	P13645	60 kDa	5	11
Leucine-rich PPR motif-containing protein, mitochondrial	LRPPRC	P42704	158 kDa	4	0
ATP synthase subunit alpha, mitochondrial	ATP5F1A	K7EK77	22 kDa	3	2
Myosin light chain 3	MYL3	P08590	22 kDa	3	2
Bovine Kappa-casein (experimentaly contaminant peptides)			21 kDa	3	2
Keratin, type I cytoskeletal 9	KRT9	P35527	62 kDa	2	8
Myosin regulatory light chain 2, skeletal muscle isoform	MYLPF	H3BML9	13 kDa	2	2
Creatine kinase M-type	CKM	P06732	43 kDa	2	3
Cluster of Histone H1.2	H1-2	P16403	21 kDa	2	1
Sarcoplasmic/endoplasmic reticulum calcium ATPase 1	ATP2A1	O14983	110 kDa	2	1
Keratin, type II cytoskeletal 6B	KRT6B	P04259	60 kDa	2	3
Fructose-bisphosphate aldolase A	ALDOA	P00883	39 kDa	2	1
T cell receptor alpha joining 56	TRAJ56	A0A075B6Z2	2 kDa	2	2
Tubulin alpha-1C chain	TUBA1C	A0A1W2PQM2	37 kDa	2	0
Destrin	DSTN	F6RFD5	15 kDa	2	1

Supplementary reference

1 Ishihama. et al. Exponentially modified protein abundance index (emPAI) for estimation of absolute protein amount in proteomics by the number of sequenced peptides per protein. Mol Cell Proteomics. 9, 1265-72, doi: 10.1074/mcp.M500061-MCP200 (2005).

Supplementary Table 3. Median replication speeds measured by a DNA fiber assay

		median(kb/min)		
	Experiment	Ctrl ASO	TUG1#1	*P* -value
HeLa/Fucci2	1	9.12	7.78	**
	2	7.16	6.06	*
	3	7.83	5.52	*
T98G	1	4.67	3.24	***
	2	7.83	5.49	*
	3	9.02	7.76	**
U251MG	1	3.65	2.63	***
	2	4.16	2.59	**
	3	3.95	2.67	***
U2OS	1	4.62	3.91	**
	2	5.89	4.12	*
	3	4.77	3.51	**

*$P > 0.05$; **$P < 0.05$; ***$P < 0.001$, Mann-Whitney U test.

Supplementary Table 4. Genomic annotation of peaks differentially altered by TUG1 KD or CPT treatment, defined by homer

Annotation	TUG1_sensitive		CPT_sensitive	
	Number of	Ratio (%)	Number of	Ratio (%)
5'UTR	0	0	32	0.168919
Promoter	10	0.94697	345	1.821157
TTS	8	0.757576	274	1.446368
Exon	6	0.568182	173	0.913218
Intron	500	47.34848	10876	57.41132
3'UTR	2	0.189394	161	0.849873
Intergenic	526	49.81061	7039	37.15688
pseudo	0	0	7	0.036951
ncRNA	4	0.378788	36	0.190034
miRNA	0	0	1	0.005279
Total	1056	100	18944	100

Supplementary Table 5. List of Abbreviations

RS	replication stress
ssDNA	single-stranded DNA
RNase H	Ribonuclease H
PIF1	Petite Integration Frequency 1
SETX	Sen1/Senataxin
FANCM	Fanconi anemia complementation group M
DHX9	DExH-Box Helicase 9
RPA	replication protein A
DSBs	double-strand breaks
lncRNAs	long noncoding RNAs
miRNAs	microRNAs
TUG1	taurine upregulated gene 1
HU	hydroxyurea
CPT	camptothecin
RT-qPCR	Reverse transcription quantitative PCR
smFISH	Single-molecule fluorescent *in situ* hybridization
DRB	5,6-dichloro-1-β-D-ribofuranosylbenzimidazole
pRPA32	phosphorylated RPA32
RIP	RNA immunoprecipitation
PCNA	proliferating cell nuclear antigen
HPRT	hypoxanthine phosphoribosyltransferase,
EdU	5-ethynyl-2'-deoxyuridine
UV	Ultraviolet
CLIP-qPCR	UV-crosslinking immunoprecipitation and qPCR
CARPID	CRISPR-assisted RNA-protein interaction detection method
ASO	antisense oligonucleotides
KD	knockdown
IdU	5-iodo-2’-deoxyuridine
CldU	5-chloro-2’-deoxyuridine
Dox	Doxycycline
rtTA	reverse tetracycline transactivator

CFP	cyan fluorescent protein
PCP	PP7-binding protein
DRIP-seq	DNA:RNA immunoprecipitation coupled with high-throughput DNA sequencing
FCM	immunohistochemistry and flow cytometry
TMZ	Temozolomide
antiTUG1-DDS	TUG1 ASO coupled with a tumor-specific drug delivery system
DDS	drug delivery system
MSI-H	microsatellite instability
ICI	immune checkpoint inhibitors
ATCC	The American Type Culture Collection
DMEM	Dulbecco's modified Eagle's medium
FBS	fetal bovine serum
Anti-anti	antibiotic-antimycotic
MSS	microsatellite stable
IC50	inhibitory concentration
CI	combination index
YBC	Y-shaped block catiomer
MGMT	O6-methylguanine-DNA methyltransferase
HE	hematoxylin and eosin
