# Supplementary material for: TUG1-mediated R-loop resolution at microsatellite loci as a prerequisite for cancer cell proliferation
Source: Nat Commun. 2023 Aug 22;14:4521. doi: 10.1038/s41467-023-40243-8 (PMC10444773; doi:10.1038/s41467-023-40243-8)
Supplement: Supplementary file 3 — Description of Additional Supplementary Files [file 41467_2023_40243_MOESM3_ESM.pdf]

Description of Additional Supplementary Files

File Name: Supplementary Data 1
Description: LncRNAs commonly upregulated by HU and CPT treatment

File Name: Supplementary Data 2
Description: List of reagents used in this study.
