# Supplementary material for: TUG1-mediated R-loop resolution at microsatellite loci as a prerequisite for cancer cell proliferation
Source: Nat Commun. 2023 Aug 22;14:4521. doi: 10.1038/s41467-023-40243-8 (PMC10444773; doi:10.1038/s41467-023-40243-8)
Supplement: Supplementary file 6 — Reporting Summary [file 41467_2023_40243_MOESM6_ESM.pdf]

nature portfolio

Corresponding author(s): Yutaka Kondo

Last updated by author(s): June 28, 2023

Reporting Summary

Nature Portfolio wishes to improve the reproducibility of the work that we publish. This form provides structure for consistency and transparency in reporting. For further information on Nature Portfolio policies, see our Editorial Policies and the Editorial Policy Checklist.

Statistics

For all statistical analyses, confirm that the following items are present in the figure legend, table legend, main text, or Methods section.

n/a	Confirmed	
☐	☒	The exact sample size (n) for each experimental group/condition, given as a discrete number and unit of measurement
☐	☒	A statement on whether measurements were taken from distinct samples or whether the same sample was measured repeatedly
☐	☒	The statistical test(s) used AND whether they are one- or two-sided<br>*Only common tests should be described solely by name; describe more complex techniques in the Methods section.*
☒	☐	A description of all covariates tested
☒	☐	A description of any assumptions or corrections, such as tests of normality and adjustment for multiple comparisons
☐	☒	A full description of the statistical parameters including central tendency (e.g. means) or other basic estimates (e.g. regression coefficient) AND variation (e.g. standard deviation) or associated estimates of uncertainty (e.g. confidence intervals)
☐	☒	For null hypothesis testing, the test statistic (e.g. F, t, r) with confidence intervals, effect sizes, degrees of freedom and P value noted<br>*Give P values as exact values whenever suitable.*
☒	☐	For Bayesian analysis, information on the choice of priors and Markov chain Monte Carlo settings
☒	☐	For hierarchical and complex designs, identification of the appropriate level for tests and full reporting of outcomes
☒	☐	Estimates of effect sizes (e.g. Cohen's d, Pearson's r), indicating how they were calculated

Our web collection on statistics for biologists contains articles on many of the points above.

Software and code

Policy information about availability of computer code

Data collection	Microarray data were acquired using Feature Extraction software, version 12.0 (Agilent Technologies). qPCR data was acquired using StepOne Software 2.3 (Thermo Fisher Scientific). smFISH data were collected using HCS Studio Cellomics Scan Version 6.6.0 (Thermo Fisher Scientific). FCM data were collected with Kaluza for Gallios 2.0 (Beckman Coulter). DNA fiber and comet assay images were acquired using LAS X 3.3 (Leica). MSI data were collected using ABI PRISM 310 Data Collection 1.1 (Applied Biosystems).
Data analysis	Half maximal inhibitory concentration (IC50) values were calculated using GraphPad Prism 8 (GraphPad Software). Combination index (CI) values were calculated using CompuSyn 1.0 (ComboSyn). Microarray data analysis was performed on GeneSpring GX, version 7.3.1 (Agilent Technologies). For all the microscopy image analysis performed in this study, ImageJ ImageJ (Fiji 2.9.0) was used. MSI was analyzed using GeneMapper v4.1 (Applied Biosystems). For FCM analysis, FlowJo software 10.6.1 (BD) was used. ModFit LT 5.0 (Verity Software House) was used for cell cycle analysis. Comet assay was analyzed on Comet Assay IV software (Perceptive Instruments). Cell proliferation data was analyzed using GraphPad Prism 9.4.1 software. MRI data were analyzed on Horos 3.0 software (Horos Project).

For manuscripts utilizing custom algorithms or software that are central to the research but not yet described in published literature, software must be made available to editors and reviewers. We strongly encourage code deposition in a community repository (e.g. GitHub). See the Nature Portfolio guidelines for submitting code & software for further information.

Data

Policy information about availability of data

All manuscripts must include a data availability statement. This statement should provide the following information, where applicable:

- Accession codes, unique identifiers, or web links for publicly available datasets
- A description of any restrictions on data availability
- For clinical datasets or third party data, please ensure that the statement adheres to our policy

The DRIP-seq data generated in this study have been deposited in the Gene Expression Omnibus (GEO) under accession code DRA013393 [https://ddbj.nig.ac.jp/resource/sra-submission/DRA013393]. The microarray data and analyzed DRIP-seq data have been deposited in the Genomic Expression Archive (GEA) under accession codes E-GEAD-362 [https://ddbj.nig.ac.jp/public/ddbj_database/gea/experiment/E-GEAD-000/E-GEAD-362/] and E-GEAD-488 [https://ddbj.nig.ac.jp/public/ddbj_database/gea/experiment/E-GEAD-000/E-GEAD-488/], respectively. The human cancer data from the cancer genome atlas (TCGA) are derived from GEPIA (Gene Expression Profiling Interactive Analysis) [http://gepia.cancer-pku.cn/]. The authors declare that all data supporting this study are available within the article, its Supplementary Information file, or the Source Data file. Source data are provided with this paper.

Research involving human participants, their data, or biological material

Policy information about studies with human participants or human data. See also policy information about sex, gender (identity/presentation), and sexual orientation and race, ethnicity and racism.

Reporting on sex and gender	No human research participants are included in this research.
Reporting on race, ethnicity, or other socially relevant groupings	See above
Population characteristics	See above
Recruitment	See above
Ethics oversight	See above

Note that full information on the approval of the study protocol must also be provided in the manuscript.

Field-specific reporting

Please select the one below that is the best fit for your research. If you are not sure, read the appropriate sections before making your selection.

☒ Life sciences ☐ Behavioural & social sciences ☐ Ecological, evolutionary & environmental sciences

For a reference copy of the document with all sections, see nature.com/documents/nr-reporting-summary-flat.pdf

Life sciences study design

All studies must disclose on these points even when the disclosure is negative.

Sample size	No statistical methods were used to predetermine sample sizes for any experiments. For in vivo and in vitro studies, sample size was selected based on pilot studies.
Data exclusions	No data were excluded from analysis.
Replication	All experiments presented in this study were performed at least twice under independent experimental conditions. All attempts at replication were successful.
Randomization	For the in vivo studies, animals were assigned randomly to experimental and control groups. The rest of the experiments were not randomized, since there was no allocation into subgroups.
Blinding	Investigators were not blinded to the experiment, since there was no allocation into subgroups.

Reporting for specific materials, systems and methods

We require information from authors about some types of materials, experimental systems and methods used in many studies. Here, indicate whether each material, system or method listed is relevant to your study. If you are not sure if a list item applies to your research, read the appropriate section before selecting a response.

Materials & experimental systems

n/a	Involved in the study
☐	☒ Antibodies
☐	☒ Eukaryotic cell lines
☒	☐ Palaeontology and archaeology
☐	☒ Animals and other organisms
☒	☐ Clinical data
☒	☐ Dual use research of concern
☒	☐ Plants

Methods

n/a	Involved in the study
☐	☒ ChIP-seq
☐	☒ Flow cytometry
☒	☐ MRI-based neuroimaging

Antibodies

Antibodies used

Rabbit monoclonal anti-E2F1, Abcam, ab179445, EPR3818(3), 2μg, ChIP
Rabbit polyclonal anti-E2F6, Abcam, ab53061, 2μg, ChIP
Rabbit monoclonal anti-cMyc, Cell Signaling Technology, 5605, D84C12, 1:500, ChIP
Rabbit IgG, MBL, PM035, 2μg, ChIP
Mouse monoclonal anti-α-Tubulin , Abcam, ab64503, DM1A, 1:1000, WB
Mouse monoclonal anti-snRNP70, Santa Cruz, sc-390899, C-3, 1:100, WB
Rabbit monoclonal anti-Histone H3, Cell signaling Technology, 4499, D1H2, 1:2000, WB
Rabbit polyclonal anti-phospho-Chk1 (Ser345) , Cell signaling Technology, 2341, 1:500, WB
Mouse monoclonal anti-Chk1, Santa Cruz, sc-8408, G-4, 1:500, WB
Rabbit polyclonal anti-phospho ATR (Thr1989), CST, 58014S, 1:500, WB
Rabbit polyclonal anti-ATR, CST, 2790S, 1:1000, WB
Rabbit monoclonal anti-GAPDH, Cell signaling Technology, 2118, 14C10, 1:1000, WB
Rabbit monoclonal anti-E2F1, Abcam, ab179445, EPR3818(3), 1:1000, WB
Rabbit polyclonal anti-E2F6, Abcam, ab53061, 1:1000, WB
Mouse monoclonal anti-β-Actin, Cell signaling Technology, 3700, 8H10D10, 1:1000, WB
Rabbit polyclonal anti-RNA Helicase A (DHX9), Abcam, ab26271, 1:1000, WB
Rabbit polyclonal anti-RPA32, BETHYL, A300-244A-M, 1:1000, WB
Mouse monoclonal anti-RPA70, Calbiochem, NA13, RPA70-9, 1:100, WB
Rabbit polyclonal anti-HA-tag, MBL, 561, 1:1000, WB
Horse anti-mouse IgG, secondary antibody, HRP, Cell signaling Technology, 7076, 1:2000, WB
Horse anti-rabbit IgG, secondary antibody, HRP, Cell signaling Technology, 7074, 1:2000, WB
Rabbit polyclonal anti-phospho-Histone H2A.X (Ser139) , Abcam, ab2893, 1:1000, WB
Rabbit monoclonal anti-phospho-Histone H2A.X (Ser139) , Abcam, ab81299, EP854(2)Y, 1:5000, WB
Rabbit polyclonal anti-phospho RPA32 (Ser33), BETHYL, A300-246A-M, 1:1000, WB
Rabbit monoclonal anti-phospho Chk2 (Thr68) , CST, 2197S, C13C1, 1:1000, WB
Rabbit monoclonal anti-Chk2, CST, 6334S, D9C6, 1:1000, WB
Rabbit monoclonal anti-phospho ATM (Ser1981) , CST , 5883S, D6H9, 1:1000, WB
Rabbit monoclonal anti-ATM, Abcam, ab201022, EPR20100, 1:1000, WB
Rabbit polyclonal anti-phosho RPA32 (Ser33), BETHYL, A300-246A-M, 1:200, IF
Rabbit polyclonal anti-RNA Helicase A (DHX9), Abcam, ab26271, 1:200, IF
Rabbit polyclonal anti-PCNA, Sigma Aldrich, HPA030521, 1:100, IF
Goat anti-rabbit IgG, secondary antibody, Alexa Fluor 488, Thermo Fisher Scientific, A11008, 1:500, IF
Mouse monoclonal anti-phospho-Histone H2A.X (Ser139) , Alexa Fluor 488, Merck Millipore, 05-636-AF488, JBW301, 1:100, IF
Mouse monoclonal anti-phospho-Histone H2A.X (Ser139) , Alexa Fluor 647, Merck Millipore, 05-636-AF647, JBW301, 1:100, IF
Mouse monoclonal anti-phospho-Histone H2A.X (Ser139) , Alexa Fluor 488, Merck Millipore, 05-636-AF488, JBW301, 1:100, FACS
Mouse monoclonal anti-GFP, MBL, M048-3, 1E4, 2μg, RIP
Mouse IgG, Sigma Aldrich, I5381, 2μg, RIP
Rabbit polyclonal anti-RNA Helicase A (DHX9), Abcam, ab26271, 2μg, CLIP
Rabbit polyclonal anti-RNA Helicase A (DHX9), Abcam, ab26271, 2μg, ChIP
Rabbit polyclonal anti-phosho RPA32 (Ser33), BETHYL, A300-246A-M, 4μg, CLIP
Rabbit IgG, MBL, PM035, 2μg-4μg, CLIP
Mouse monoclonal Anti-DNA-RNA Hybrid, clone S9.6, Merck Millipore, MABE1095, S9.6, 1:1000, Slot Blot
Mouse monoclonal Anti-DNA-RNA Hybrid, clone S9.6, Merck Millipore, MABE1095, S9.6, 1:100, DRIP
Rat monoclonal anti-BrdU, Abcam, ab6326-250, BU1/75 (ICR1), 1:150, DNA fiber
Mouse monoclonal anti-BrdU, BD, 347580, B44(RUO (GMP)), 1:500, DNA fiber
Goat anti-mouse IgG, secondary antibody, Alexa Fluor 488, Thermo Fisher Scientific, A11029, 1:100, DNA fiber
Donkey anti-rat IgG, secondary antibody, Cy3, Jackson, 712-165-153, 1:400, DNA fiber

Validation

All antibodies are commercially available and validated for use in their respective applications as stated on the manufacturers' product pages:
Rabbit monoclonal anti-E2F1 Abcam ab179445 https://www.abcam.com/products/primary-antibodies/e2f1-antibody-epr38183-chip-grade-ab179445.html
Rabbit polyclonal anti-E2F6 Abcam ab53061 https://www.abcam.com/products/primary-antibodies/e2f6-antibody-ab53061.html
Rabbit monoclonal anti-cMyc Cell Signaling Technology 5605 https://www.cellsignal.com/products/primary-antibodies/c-myc-d84c12-rabbit-mab/5605
Mouse monoclonal anti-α-Tubulin Abcam ab64503 https://www.abcam.com/products/primary-antibodies/fitc-alpha-tubulin-antibody-dm1a-microtubule-marker-ab64503.html
Mouse monoclonal anti-snRNP70 Santa Cruz sc-390899 https://www.scbt.com/p/u1-snrnp-70-antibody-c-3?requestFrom=search

Rabbit monoclonal anti-Histone H3 Cell signaling Technology 4499 https://www.cellsignal.jp/products/primary-antibodies/histone-h3-d1h2-xp-rabbit-mab/4499
Rabbit polyclonal anti-phospho-Chk1 (Ser345) Cell signaling Technology 2341 https://www.cellsignal.jp/products/primary-antibodies/phospho-chk1-ser345-antibody/2341
Mouse monoclonal anti-Chk1 Santa Cruz sc-8408 https://www.scbt.com/p/chk1-antibody-g-4?requestFrom=search
Rabbit polyclonal anti-phospho ATR (Thr1989) CST 58014S https://www.cellsignal.jp/products/primary-antibodies/phospho-atr-thr1989-antibody/58014?site-search-type=Products&N=4294956287&Ntt=58014s&fromPage=plp&_requestid=3298946
Rabbit polyclonal anti-ATR CST 2790S https://www.cellsignal.jp/products/primary-antibodies/atr-antibody/2790?site-search-type=Products&N=4294956287&Ntt=2790s%C2%A0%C2%A0%C2%A0%C2%A0%C2%A0%C2%A0%C2%A0%C2%A0%C2%A0+&fromPage=plp&_requestid=3299022
Rabbit monoclonal anti-GAPDH Cell signaling Technology 2118 https://www.cellsignal.jp/products/primary-antibodies/gapdh-14c10-rabbit-mab/2118
Mouse monoclonal anti-β-Actin Cell signaling Technology 3700 https://www.cellsignal.jp/products/primary-antibodies/b-actin-8h10d10-mouse-mab/3700
Rabbit polyclonal anti-RNA Helicase A (DHX9) Abcam ab26271 https://www.abcam.com/products/primary-antibodies/rna-helicase-a-antibody-ab26271.html
Rabbit polyclonal anti-RPA32 BETHYL A300-244A-M https://www.fortislife.com/products/primary-antibodies/rabbit-anti-rpa32-antibody/BETHYL-A300-244
Mouse monoclonal anti-RPA70 Calbiochem NA13 https://www.merckmillipore.com/JP/ja/product/Anti-Replication-Protein-A-Ab-1-Mouse-mAb-RPA70-9,EMD_BIO-NA13
Rabbit polyclonal anti-HA-tag MBL 561 https://ruo.mbl.co.jp/bio/e/dtl/A/?pcd=561
Rabbit polyclonal anti-phospho-Histone H2A.X (Ser139) Abcam ab2893 https://www.abcam.com/products/primary-antibodies/gamma-h2ax-phospho-s139-antibody-ab2893.html
Rabbit monoclonal anti-phospho-Histone H2A.X (Ser139) Abcam ab81299 https://www.abcam.com/products/primary-antibodies/gamma-h2ax-phospho-s139-antibody-ep8542y-ab81299.html
Rabbit polyclonal anti-phospho RPA32 (Ser33) BETHYL A300-246A-M https://www.fortislife.com/products/primary-antibodies/rabbit-anti-phospho-rpa32-s33-antibody/BETHYL-A300-246
Rabbit monoclonal anti-phospho Chk2 (Thr68) CST 2197S https://www.cellsignal.jp/products/primary-antibodies/phospho-chk2-thr68-c13c1-rabbit-mab/2197?site-search-type=Products&N=4294956287&Ntt=2197s&fromPage=plp&_requestid=3299099
Rabbit monoclonal anti-Chk2 CST 6334S https://www.cellsignal.jp/products/primary-antibodies/chk2-d9c6-rabbit-mab/6334?site-search-type=Products&N=4294956287&Ntt=6334s&fromPage=plp&_requestid=3299139
Rabbit monoclonal anti-phospho ATM (Ser1981) CST 5883S https://www.cellsignal.jp/products/primary-antibodies/phospho-atm-ser1981-d6h9-rabbit-mab/5883?site-search-type=Products&N=4294956287&Ntt=5883s&fromPage=plp&_requestid=3299198
Rabbit monoclonal anti-ATM Abcam ab201022 https://www.abcam.com/products/primary-antibodies/atm-antibody-epr20100-chip-grade-ab201022.html
Rabbit polyclonal anti-PCNA Sigma Aldrich HPA030521 https://www.sigmaaldrich.com/JP/ja/product/sigma/hpa030521
Mouse monoclonal anti-phospho-Histone H2A.X (Ser139) , Alexa Fluor 488 Merck Millipore 05-636-AF488 https://www.merckmillipore.com/JP/ja/product/Anti-phospho-Histone-H2A.X-Ser139-clone-JBW301-Alexa-Fluor-488-Conjugate-Antibody,MM_NF-05-636-AF488
Mouse monoclonal anti-phospho-Histone H2A.X (Ser139) , Alexa Fluor 647 Merck Millipore 05-636-AF647 https://www.merckmillipore.com/JP/ja/product/Anti-phospho-Histone-H2A.X-Ser139-Antibody-clone-JBW301-Alexa-Fluor-647,MM_NF-05-636-AF647
Mouse monoclonal anti-GFP MBL M048-3 https://ruo.mbl.co.jp/bio/e/dtl/A/?pcd=M048-3
Mouse monoclonal Anti-DNA-RNA Hybrid, clone S9.6 Merck Millipore MABE1095 https://www.merckmillipore.com/JP/ja/product/Anti-DNA-RNA-Hybrid-Antibody-clone-S9.6,MM_NF-MABE1095
Rat monoclonal anti-BrdU Abcam ab6326-250 https://www.abcam.com/products/primary-antibodies/brdu-antibody-bu175-icr1-proliferation-marker-ab6326.html
Mouse monoclonal anti-BrdU BD 347580 https://www.bdbiosciences.com/ja-jp/products/reagents/flow-cytometry-reagents/clinical-discovery-research/single-color-antibodies-ruo-gmp/purified-mouse-anti-brdu.347580

Eukaryotic cell lines

Policy information about cell lines and Sex and Gender in Research

Cell line source(s)	Cell lines were obtained from ATCC (LN229, CRL-2611; U2OS, HTJaB-96), RIKEN Cell Bank (HeLa/Fucci2, RCB2867; HeLa, RCB0007; TIG3, RCB4468; HEK293T,RCB2202), or JCRB Cell Bank (U251MG, IFO50288). U2OS 2-6-3 was obtained from the laboratory of David L. Spector at Cold Spring Harbor Laboratory (Janicki et al., Cell, 2004).
Authentication	Cell lines were authenticated by their distributers, ATCC, RIKEN Cell Bank, and JCRB via STR profiling. U2OS 2-6-3 cells was not authenticated, however these cells have been used in numerous studies. For instance:<br>Kumaran and Spector, J Cell Biol, 2008; Newhart et al., J Biol Chem, 2013, Normanno et al., Nature Commun, 2015; Wei et al., PNAS, 2015; Luijsterburg et al., Mol Cell, 2016.
Mycoplasma contamination	Cell lines were routinely tested for mycoplasma contamination using EZ-PCR Mycoplasma Detection Kit (Biological Industries). All cell cultures used in this study tested negative to Mycoplasma contamination.
Commonly misidentified lines (See ICLAC register)	None of the cell lines used in this study are listed in the ICLAC register of commonly misidentified cell lines.

Animals and other research organisms

Policy information about studies involving animals; ARRIVE guidelines recommended for reporting animal research, and Sex and Gender in Research

Laboratory animals	NOD.CB17-Prkdcscid /J (NOD SCID) (The Jackson Laboratory Japan, RRID:IMSR_JAX:001303)<br>Approximately 6-week-old female mice were used in the xenograft mouse brain tumor model and treatment experiments. Mice were housed under standard, regulated conditions; 12/12 light/dark cycle, temperature at 21°C ± 4°C and humidity 40-70%.
Wild animals	No wild animals were used in this study.
Reporting on sex	Female NOD SCID mice were used in the xenograft studies.
Field-collected samples	No field-collected samples were used in this study.
Ethics oversight	All experiments using live animals were reviewed and approved by the Animal Care and Use Committee of Nagoya University Graduate School of Medicine (approval number 20271).

Note that full information on the approval of the study protocol must also be provided in the manuscript.

Plants

Seed stocks	No plant research is included in this study.
Novel plant genotypes	See above
Authentication	See above

ChIP-seq

Data deposition

☒ Confirm that both raw and final processed data have been deposited in a public database such as GEO.

☒ Confirm that you have deposited or provided access to graph files (e.g. BED files) for the called peaks.

Data access links<br>*May remain private before publication.*	GEA reviewer access system: https://ddbj.nig.ac.jp/gea/reviewer/login<br>Experiment Accession: E-GEAD-488, Access key: VGVgVbLbpOFj0X0pCCpQ
Files in database submission	DRIP_HeLa_Cont_DMSO_r1.hg19.rpm.bw<br>DRIP_HeLa_Cont_DMSO_r2.hg19.rpm.bw<br>DRIP_HeLa_Cont_DMSO_r3.hg19.rpm.bw<br>DRIP_HeLa_Cont_CPT_r1.hg19.rpm.bw<br>DRIP_HeLa_Cont_CPT_r2.hg19.rpm.bw<br>DRIP_HeLa_Cont_CPT_r3.hg19.rpm.bw<br>DRIP_HeLa_kdTug1_DMSO_r1.hg19.rpm.bw<br>DRIP_HeLa_kdTug1_DMSO_r2.hg19.rpm.bw<br>DRIP_HeLa_kdTug1_DMSO_r3.hg19.rpm.bw<br>DRIP_HeLa_kdTug1_CPT_r1.hg19.rpm.bw<br>DRIP_HeLa_kdTug1_CPT_r2.hg19.rpm.bw<br>DRIP_HeLa_kdTug1_CPT_r3.hg19.rpm.bw<br>CPT_sensitive.bed<br>Tug1_sensitive.bed
Genome browser session<br>(e.g. UCSC)	Not applicable

Methodology

Replicates	DRIP-seq experiments were performed in three biological replicates.
Sequencing depth	Libraries were sequenced (2x150 bp) on a Illumina Hiseq 2500 platform (Illumina)
Antibodies	Mouse monoclonal Anti-DNA-RNA Hybrid, clone S9.6 (Merck Millipore, MABE1095)
Peak calling parameters	The sequencing reads were mapped to hg19 using STAR (version 2.5.3). Duplicated reads were then removed using MarkDuplicates.jar (Picard version 1.29), Peak-calling (MACS2, version 2.2.7.1) and IDR (irreproducible discovery rate, ide version 2.0.3) analyses were performed according to the ENCODE guidelines62,63. Briefly, peak-calling was performed with a less stringent p-value threshold (1e-3), and peak consistency was evaluated based on signal values with a 1% threshold. The ratio between the number of peaks consistent between true replicates (Nt) and between pooled pseudoreplicates (Np) was calculated in all the

combinations. When all the combinations among three replicates satisfy Np/Nt <2, this indicates reliable replicates. Peaks consistently satisfying the above criteria among replicates were used for downstream analyses.

Data quality	The quality of the fastq files was checked using the FastQC software.
Software	STAR (version 2.5.3), MarkDuplicates.jar (Picard version 1.29), Peak-calling (MACS2, version 2.2.7.1), IDR (irreproducible discovery rate, ide version 2.0.3), DiffBind (version 3.0.15), homer (version 4.11.1), deepTools (version 3.5.1), and Bedtools (version 2.30.0) were used.

Flow Cytometry

Plots

Confirm that:

☒ The axis labels state the marker and fluorochrome used (e.g. CD4-FITC).

☒ The axis scales are clearly visible. Include numbers along axes only for bottom left plot of group (a 'group' is an analysis of identical markers).

☒ All plots are contour plots with outliers or pseudocolor plots.

☒ A numerical value for number of cells or percentage (with statistics) is provided.

Methodology

Sample preparation	Cells were washed with PBS and incubated for 15 min on ice in hybridization buffer (PBS containing 1.0% BSA and 0.25% TritonX-100). After centrifugation, cells were hybridized with an anti-γ-H2AX antibody (Supplementary Table S3) for 1 h in the dark at room temperature (24-26 °C). Cells were then stained with FxCycle™ Violet Stain (Thermo Fisher Scientific) for 30 min before FCM. An apoptosis assay was conducted by using Annexin V-FITC Apoptosis Detection Kit (Nacalai Tesque). For analysis of EdU incorporation into newly synthesized DNA, cells were incubated with 10 µM EdU for 1 h and then processed using Click-iT™ Plus EdU Alexa Fluor™ 647 Flow Cytometry Assay Kit (Thermo Fisher Scientific), according to the manufacturer's instructions.
Instrument	Beckman Coulter Gallios Flow Cytometer
Software	The data were analyzed using FlowJo software 10.6.1 (BD, NJ, USA). The percentage of each cell cycle population was analyzed by ModFit LT 5.0 (Verity Software House, ME, USA).
Cell population abundance	A minimum of 10,000 events were acquired per sample following debris and doublet exclusion.
Gating strategy	Initial cell populations were gated using FSC and SSC plot of cell only (unstained) control sample to remove cell debris, except in protocols where apoptotic populations were measured. Doublets and cell aggregates were excluded by gating in single cells. The cell population gated in after debris and doublet exclusion was then used to create single-staining histograms (cell cycle based on FxCycle Violet Stain and DNA damage based on anti-gamma-H2AX antibody) and double-staining quadrants (PI and anti-annexin V-FITC for apoptosis assays).

☒ Tick this box to confirm that a figure exemplifying the gating strategy is provided in the Supplementary Information.
